# Comparative Efficacy of Systemic Agents for Brain Metastases From Non-Small-Cell Lung Cancer With an EGFR Mutation/ALK Rearrangement: A Systematic Review and Network Meta-Analysis

**DOI:** 10.3389/fonc.2021.739765

**Published:** 2021-12-07

**Authors:** Shervin Taslimi, Karanbir Brar, Yosef Ellenbogen, Jiawen Deng, Winston Hou, Fabio Y. Moraes, Michael Glantz, Brad E. Zacharia, Aaron Tan, Manmeet S. Ahluwalia, Mustafa Khasraw, Gelareh Zadeh, Alireza Mansouri

**Affiliations:** ^1^ Division of Neurosurgery, Department of Surgery, Queen’s University, Kingston, ON, Canada; ^2^ Faculty of Medicine, University of Toronto, Toronto, ON, Canada; ^3^ Faculty of Health Sciences, McMaster University, Hamilton, ON, Canada; ^4^ Department of Oncology, Queen’s University, Kingston, ON, Canada; ^5^ Department of Neurosurgery, Penn State Health, Hershey, PA, United States; ^6^ Penn State Cancer Institute, Hershey, PA, United States; ^7^ Division of Medical Oncology, National Cancer Center Singapore, Singapore, Singapore; ^8^ Rose Ella Burkhardt Brain Tumor and Neuro-Oncology Center, Taussig Cancer Institute, Cleveland Clinic, Cleveland, OH, United States; ^9^ Department of Hematology/Oncology, Taussig Cancer Institute, Cleveland Clinic, Cleveland, OH, United States; ^10^ The Preston Robert Tisch Brain Tumor Center, Duke University, Durham, NC, United States

**Keywords:** targeted therapy, brain metastases, non-small cell lung cancer, neuro-oncology, EGFR inhibitors, ALK inhibitors

## Abstract

**Background:**

Brain metastases (BM) from non-small-cell lung cancer (NSCLC) are frequent and carry significant morbidity, and current management options include varying local and systemic therapies. Here, we performed a systematic review and network meta-analysis to determine the ideal treatment regimen for NSCLC BMs with targetable EGFR-mutations/ALK-rearrangements.

**Methods:**

We searched MEDLINE, EMBASE, Web of Science, ClinicalTrials.gov, CENTRAL and references of key studies for randomized controlled trials (RCTs) published from inception until June 2020. Comparative RCTs including ≥10 patients were selected. We used a frequentist random-effects model for network meta-analysis (NMA) and assessed the certainty of evidence using the GRADE approach. Our primary outcome of interest was intracranial progression-free survival (iPFS).

**Results:**

We included 24 studies representing 19 trials with 1623 total patients. Targeted tyrosine kinase inhibitors (TKIs) significantly improved iPFS, with second-and third- generation TKIs showing the greatest benefit (HR=0.25, 95%CI 0.15-0.40). Overall PFS was also improved compared to conventional chemotherapy (HR=0.47, 95%CI 0.36-0.61). In EGFR-mutant patients, osimertinib showed the greatest benefit in iPFS (HR=0.32, 95%CI 0.15-0.69) compared to conventional chemotherapy, while gefitinib + chemotherapy showed the greatest overall PFS benefit (HR=0.26, 95%CI 0.10-0.70). All ALKi improved overall PFS compared to conventional chemotherapy, with alectinib having the greatest benefit (HR=0.13, 95%CI 0.07-0.24).

**Conclusions:**

In patients with NSCLC BMs and EGFR/ALK mutations, targeted TKIs improve intracranial and overall PFS compared to conventional modalities such as chemotherapy, with greater efficacy seen using newer generations of TKIs. This data is important for treatment selection and patient counseling, and highlights areas for future RCT research.

**Systematic Review Registration:**

https://www.crd.york.ac.uk/prospero/display_record.php?RecordID=179060.

## Introduction

Non-small cell lung cancer (NSCLC) is one of the most common and lethal cancer subtypes, with 25-30% of patients developing brain metastases (BMs) over the course of their disease (1). While surgery and radiation-based therapies have been the mainstay of management for local disease control in the brain (2–5), the emergence of targeted therapeutics based on the molecular features of tumors – such as tyrosine kinase inhibitors (TKIs) - have expanded our therapeutic armamentarium. Whereas traditional chemotherapeutic regimens have had limited efficacy against BMs (6), partly perhaps due to the inability to cross the blood-brain barrier (BBB), TKIs have shown significant promise in the management of people with NSCLC BM harboring targetable mutations in several clinical trials (3, 4, 7–9). In particular, newer generations of TKI have been developed to improve BBB penetrance and overcome resistance that has developed to earlier generations, improving their efficacy.

Despite convincing randomized controlled trial (RCT) data, however, to date there has been no comprehensive pooled analysis of the efficacy of the various generations of TKIs in comparison to traditional therapies for BMs, including systemic chemotherapy combined with other local therapies. The emergence of newer generations of TKIs, their individual side effect profiles, and their potentially prohibitive cost, necessitates assessment of their comparative efficacy in order to provide physicians with clinically relevant data that can aid decision-making and provide comprehensive patient counseling. However, head-to-head comparisons in the setting of an RCT are limited.

A network meta-analysis (NMA) allows for comparisons of multiple interventions, particularly when direct comparisons between interventions may be lacking (10). As such, we performed a systematic review and NMA to compare the efficacy of the various targeted therapies, compared with conventional chemotherapy and radiotherapy as a reference, in patients with EGFR mutated or ALK rearranged NSCLC BMs.

## Methods

This study was performed based on a predefined protocol and in accordance with the Preferred Reporting Items for Systematic Reviews and Meta-Analyses (PRISMA) Extension statement for reporting on network meta-analyses. This study is registered with the International Prospective Register of Systematic Reviews (PROSPERO), ID CRD42020179060.

### Search Strategy

We searched MEDLINE, Embase, Cochrane Controlled Register of Trials (CENTRAL), and Web of Science from inception until June 2020 for RCTs. We also searched the grey literature including ClinicalTrials.gov, as well as references of included papers and past review articles. We utilized filters to select for RCTs and human studies wherever possible. We did not restrict results by language. Search terms included “brain metastases”, “immunotherapy”, “targeted therapy”, “surgery, “radiosurgery”, and “chemotherapy.” A full set of search terms and strategies for each database can be found in Supplement A.

### Study Selection and Eligibility Criteria

All studies were screened independently and in duplicate by KB, JD, YE, and WH. Our study was designed using the PICOS method, as outlined in detail in the following sections. Our population included all adults with NSCLC with either an activating EGFR or ALK mutation, with one or more BM confirmed *via* imaging (CT/MRI). We included all RCTs independent of language with ≥10 patients, that compared at least two independent treatment regimens for EGFR mutant or ALK rearranged NSCLC and reported data on patients with BMs. Foreign language studies were translated to English.

### Data Extraction and Quality Assessment

Data were extracted independently and in duplicate, using a standardized form. We sought to contact primary authors for missing data where possible. Pre-specified variables of interest included design-related variables, phase, eligibility criteria, intervention arms and descriptions, performance status (KPS or ECOG), duration of treatment and follow-up, and patient demographics (age [median, range], sex).

Our primary outcome was intracranial progression free survival (iPFS), with secondary outcomes including overall PFS, overall survival (OS), intracranial time to progression (iTTP, defined as the time from randomization to disease progression in the brain), and adverse reactions. Many NSCLC clinical trials have excluded patients with BMs or the main outcomes of interest have not included the response of BMs to therapy. Furthermore, most individuals with metastatic disease succumb to their systemic tumor burden. Therefore, we selected iPFS as the primary outcome in order to focus on the efficacy of any given treatment on the burden of intracranial disease, without confounding from the primary cancer. We only included studies that reported a comparative hazard ratio (HR) between arms for each outcome; the raw median survival times were not used in the analysis.

We performed quality assessment of the included studies using the Cochrane Risk of Bias 2.0 tool (11). Two analysts completed risk of bias assessment in duplicate, and disagreements were resolved *via* consensus. We used CiNEMA, a novel GRADE-based method for assessing confidence in results when multiple interventions are compared, to assess the overall certainty of evidence associated with each analysis (12, 13).

### Data Synthesis and Statistical Analysis

A fixed effects or random effect meta-analysis was planned to compare the overall effect of targeted therapy with conventional chemotherapeutic agents for primary and secondary outcomes. We then performed a planned subgroup analysis for EGFR mutated and ALK re-arranged patients. For each outcome, we used HR and calculated the corresponding standard error (SE) for all analyses. In each subgroup, to compare different treatments, we used a frequentist NMA. This approach synthesizes metrics of both direct and indirect comparisons to refine and generate estimates of all possible pair-wise comparisons within a network. When both direct and indirect evidence of a comparison between treatment modalities were available, we first tested the null hypothesis that direct and indirect estimates were similar when enough information was available. When the null hypothesis was not rejected, the treatment effect was synthesized together to yield a network treatment effect. We then used the R̈cker & Schwarzer method to rank treatments (14). We combined similar treatments into single nodes where necessary to complete the analysis. In particular, we combined most traditional chemotherapeutic regimens into a single node for most analyses, as various combination approaches have been shown to be similarly efficacious to traditional monotherapy in large trials (15, 16). Where necessary, we grouped EGFR inhibitors (EGFRi) by generation, with first generation defined as gefitinib, erlotinib, and icotinib, second generation as afatinib, and third generation as osimertinib. We also grouped ALK inhibitors (ALKi) similarly, with first generation as crizotinib, and second generation as ceritinib, alectinib, and brigatinib.

We assessed heterogeneity using Cochran’s Q statistics or the Chi square test in the case of pairwise meta-analysis. A P value of 0.1 was considered significant heterogeneity. In case of heterogeneity between studies a random effects model was used, otherwise a fixed effects model was used. A two-way P value of less than 0.05 was considered statistically significant. R software version 3.6.3 was used for all analyses.

## Results

### Search Results and Study Characteristics

**Figure 1:**
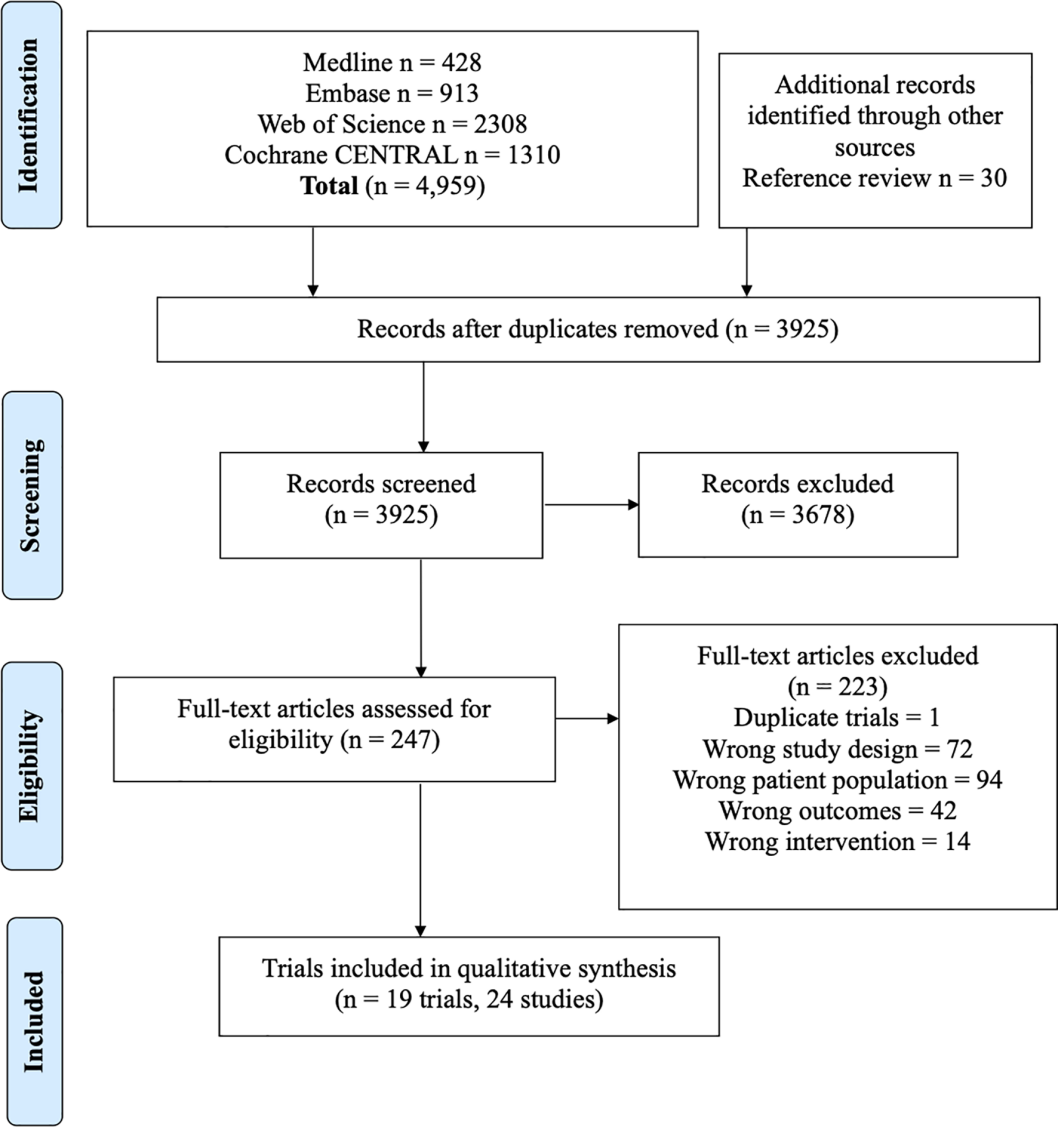
PRISMA flowchart outlining study screening process, with reasons for exclusion at full-text screening stage outlined.

Twenty-four studies were included representing 19 unique trials, with 1623 patients total (**Figure 1**). All trials included patients with favorable performance status (ECOG 0-2 or KPS>70) (7–9, 17–33). Nine trials included patients with EGFR mutations, and 10 included patients with ALK rearrangements.

Importantly, most trials that reported outcome data on BMs as a subgroup analysis of all-comer NSCLC patients excluded BMs that were symptomatic or required urgent treatment, meaning many of these patients may have been previously treated with modalities such as surgery or radiation. This was true for all included studies except for Yang 2017 (7). Baseline characteristics and extracted data from included trials are shown in **Tables 1**, **2**.

**Table 1:** Summary demographics and characteristics of included trials.

Study ID	Trial Design	Patient Population	Arm	Category of Intervention	N BM patients	N women (%)	Median age, years (range)	Previous BM treatments
Camidge et al. (17) (ALTA-1L, NCT0273750)	Phase III, Open-Label, Multicentre, international	ALK-rearranged NSCLC Asymptomatic, stable BMs only	Arm A: Brigatinib	TKI (ALK Gen 3 + EGFR Gen 3)	40	69 (50%), full cohort	58 (27-86), full cohort	Brain radiotherapy, n=18
			Arm B: Crizotinib	TKI (ALK Gen 1)	41	81 (59%), full cohort	60 (29-89) full cohort	Brain radiotherapy, n=19
Hida et al. (18) (J-ALEX, JapicCTI-132316)	Phase III, Open-Label, Multicentre, Japanese centres only	ALK-rearranged NSCLC Asymptomatic, stable BMs only	Arm A: Alectinib	TKI (ALK Gen 2)	14	62 (60%), full cohort	61 (27-85), full cohort	Brain radiotherapy, n=6/16
			Arm B: Crizotinib	TKI (ALK Gen 1)	29	63 (61%), full cohort	59.5 (25-84), full cohort	Brain radiotherapy, n=16/31
Yang (7) (BRAIN, NCT01724801)	Phase III, Open-Label, Multicentre, Chinese centres only	EGFR-mutated NSCLC	Arm A: Icotinib	TKI (EGFR Gen 1)	85	53 (62%)	57 (51-64)	No prior TKI or WBRT
			Arm B: WBRT + Platinum-based Chemotherapy	WBRT + Traditional Chemotherapy	73	41 (56%)	58 (48-63)	
Wu et al. (9) (AURA3, NCT02151981)	Phase III, Open-Label, Multicentre, international	EGFR-mutated NSCLC Stable, asymptomatic BMs only Leptomeningeal metastases excluded	Arm A: Osimertinib	TKI (EGFR Gen 3)	75	41 (55%)	58 (34-82)	Brain radiotherapy, n=28
			Arm B: Platinum-based Chemotherapy	Traditional Chemotherapy	41	29 (71%)	59 (20-79)	Brain radiotherapy, n=20
Soria et al. (19) (FLAURA, NCT02296125)	Phase III, Double-Blind, Multicentre, International	EGFR-mutated NSCLC Stable BMs only	Arm A: Osimertinib	TKI (EGFR Gen 3)	53	178 (63.8%), full cohort	64 (26-85), full cohort	No prior treatment for advanced disease, no prior treatment with TKI
			Arm B: Standard EGFR-TKI (Gefitinib or Erlotinib)	TKI (EGFR Gen 1)	63	172 (62%), full cohort	64 (35-93), full cohort	
Novello et al. (21) (ALUR, NCT02604342)	Phase III, Open-Label, Multicentre, international	ALK-rearranged NSCLC All patients had two lines of previous systemic therapy, including 1 line of previous Crizotinib therapy. Asymptomatic BMs OR symptomatic BMs and ineligible for radiotherapy only.	Arm A: Alectinib	TKI (ALK Gen 2)	47	31 (43.1%), full cohort	55.5 (21-82), full cohort	WBRT (n=23), SRS (n=2), other (n=3). All patients had previous crizotinib therapy
			Arm B: Chemotherapy (Pemetrexed OR Docetaxel)	Traditional Chemotherapy	26	18 (51.4%), full cohort	59 (37-80), full cohort	WBRT (n=9), SRS (n=5), other (n=2). All patients had previous crizotinib therapy
Peters et al. (22) (ALEX, NCT02075840)	Phase III, Open-Label, Multicentre, international	ALK-rearranged NSCLC Leptomeningeal metastases excluded Asymptomatic BMs only	Arm A: Crizotinib	TKI (ALK Gen 1)	58	87 (58%), full cohort	54 (18-91), full cohort	Surgery (n=1), SRS(n=4), WBRT (n=16), other (n=1)
			Arm B: Alectinib	TKI (ALK Gen 2)	64	84 (55%), full cohort	58 (25-88), full cohort	Surgery (n=1), SRS (n=5), WBRT (n=17), other (n=4)
Solomon et al. (23–25) (PROFILE 1014, NCT01154140)	Phase III, Open-Label, Multicentre, international	ALK-rearranged NSCLC Stable and previously treated BMs only	Arm A: Crizotinib	TKI (ALK Gen 1)	39	19 (49%)	48 (29-70)	No prior systemic treatment of advanced disease
			Arm B: Platinum-based Chemotherapy	Traditional Chemotherapy	40	31 (78%)	51 (25-76)	
Wu et al. (26) (PROFILE 1029, NCT01639001)	Phase III, Open-Label, Multicentre, Chinese centres only	ALK-rearranged NSCLC Stable and previously treated BMs only	Arm A: Crizotinib	TKI (ALK Gen 1)	21	54 (51.9%), full cohort	48 (24-67), full cohort	No previous systemic therapy for advanced disease
			Arm B: Platinum-based Chemotherapy	Traditional Chemotherapy	32	60 (58.3%), full cohort	50 (23-69), full cohort	
Zhou et al. (27) (ALESIA, NCT02838420)	Phase III, Open-Label, Multicentre, international	ALK-rearranged NSCLC All symptomatic BMs had to be previously treated with radiotherapy	Arm A: Alectinib	TKI (ALK Gen 2)	44	61, full cohort	51 (43-59), full cohort	Brain radiotherapy (n=8)
			Arm B: Crizotinib	TKI (ALK Gen 1)	23	28, full cohort	49 (41-59), full cohort	Brain radiotherapy (n=5)
Shaw et al. (28) (NCT00932893)	Phase II, Open-Label, Multicentre, International	ALK-rearranged NSCLC, all patients had previous 1 line of platinum-based therapy. Asymptomatic BMs only.	Arm A: Crizotinib	TKI (ALK Gen 1)	60	98 (56.64%), full cohort	51 (22-81), full cohort	Progression after 1 platinum-based chemotherapy regimen
			Arm B: Chemotherapy (Pemetrexed or Docetaxel)	Traditional Chemotherapy	60	96 (55.17%), full cohort	49 (24-85), full cohort	
Shaw et al. (29) (ASCEND-5, NCT01828112)	Phase III, Open-Label, Multicentre, International	ALK-rearranged NSCLC, all patients had previous platinum-based chemotherapy and crizotinib. Asymptomatic BMs only.	Arm A: Ceritinib	TKI (ALK Gen 2)	60	68 (59%), full cohort	54, full cohort	Progression after prior treatment on crizotinib + chemotherapy
			Arm B: Chemotherapy (Pemetrexed or Docetaxel)	Traditional Chemotherapy	59	61 (53%), full cohort	54 (47-64), full cohort	
Soria et al. (20) (ASCEND-4, NCT01828099)	Phase III, Open-Label, Multicentre, International	ALK-rearranged NSCLC, Stable and asymptomatic BMs only.	Arm A: Ceritinib	TKI (ALK Gen 2)	59	102 (54%), full cohort	55 (22-81), full cohort	Brain radiotherapy (n=24). Adjuvant or neoadjuvant chemo (n=10)
			Arm B: Platinum-based Chemotherapy	Traditional Chemotherapy	62	114 (62%), full cohort	54 (22-80), full cohort	Brain radiotherapy (n=26). Adjuvant or neoadjuvant chemo (n=9)
Schuler et al. (30) (LUX-Lung 3, NCT00949650)	Phase III, Open-Label, Multicentre, international	EGFR-mutated NSCLC, no prior treatment for NSCLC, no prior TKI. Stable, asymptomatic BMs only.	Arm A: Afatinib	TKI (EGFR Gen 2)	20	14 (70%)	60.5 (37-71)	WBRT (n=7)
			Arm B: Platinum-based Chemotherapy (Cisplatin/Pemetrexed)	Traditional Chemotherapy	15	12 (80%)	63 (31-74)	WBRT (n=5)
Schuler et al. (30) (LUX-Lung 6, NCT01121393)	Phase III, Open-Label, Multicentre, international (Asia only)	EGFR-mutated NSCLC, no prior treatment for NSCLC, no prior TKI. Stable, asymptomatic BMs only.	Arm A: Afatinib	TKI (EGFR Gen 2)	28	19 (67.9%)	53.5 (30-78)	WBRT (n=6).
			Arm B: Platinum-based Chemotherapy (Cisplatin/Gemcitabine)	Traditional Chemotherapy	18	12 (66.7%)	55 (35-70)	WBRT (n=6)
Park et al. (31) (LUX-Lung 7, NCT01466660.)	Phase IIB, Open-Label, Multicentre, international	EGFR-mutated NSCLC, no prior treatment for NSCLC, no prior TKI. Stable, asymptomatic BMs only.	Arm A: Afatinib	TKI (EGFR Gen 2)	26	91, full cohort	63 (30-86), full cohort	NR
			Arm B: Gefitinib	TKI (EGFR Gen 1)	24	106, full cohort	63 (32-89), full cohort	NR
Hosomi et al. (32) (NEJ009, UMIN000006340)	Phase III, Open-Label, Multicentre, Japanese centres only	EGFR-mutated NSCLC, Asymptomatic BMs only	Arm A: Gefinitib	TKI (EGFR Gen 1)	38	108, full cohort	Mean 64 (SD 8.4), full cohort	Brain radiation (n=15)
			Arm B: Gefitinib + Platinum-based Chemotherapy	TKI (EGFR Gen 1) + Traditional Chemotherapy	50	114, full cohort	Mean 64.8 (SD 7.8), full cohort	Brain radiation (n=17)
Saito et al. (33) (NEJ026, UMIN000017069)	Phase III, Open-Label, Multicentre, international	EGFR-mutated NSCLC, Asymptomatic BMs only	Arm A: Erlotinib + Bevacizumab	TKI (EGFR Gen 1) + Traditional Chemotherapy (VEGFi)	36	71 (63%), full cohort	67 (61-73), full cohort	Patients could not have received previous chemotherapy other than adjuvant chemotherapy
			Arm B: Erlotinib alone	TKI (EGFR Gen 1)	36	73 (65%), full cohort	68 (62-73), full cohort	
Noronha et al. (8) (CTRI/2016/08 /007149)	Phase III, Open-Label, Single-centre, India	EGFR-mutated NSCLC	Arm A: Gefinitib	TKI (EGFR Gen 1)	34	83 (47%), full cohort	56 (27*-*78), full cohort	WBRT (n=31)
			Arm B: Gefitinib + Platinum-based Chemotherapy	TKI Gen 1 + Traditional Chemotherapy	30	86 (49%), full cohort	54 (27-75), full cohort	WBRT (n=22)

**Table 2:** Extracted outcome data from each study.

Study ID	Treatment Arm	Overall Survival	Overall PFS (Definition)	Overall PFS (HR)	Intracranial PFS (Definition)	Intracranial PFS (HR)	Intracranial Time to Progression (Definition)	Intracranial TTP (HR)
Camidge et al. (17) (ALTA-1L, NCT0273750)	Arm A: Brigatinib	NR	NR	NR	Time from randomization to CNS disease progression based on RECIST v1.1 criteria, or death from any cause	0.27 (0.13-0.54)	NR	NR
	Arm B: Crizotinib					Reference		
Hida et al. (18) (J-ALEX, JapicCTI-132316)	Arm A: Alectinib	NR	NR	NR	Time to progression of BMs in patients with BMs at baseline, or death, progression based on RECIST v.1.1 criteria	0.16 (0.02-1.28)	NR	NR
	Arm B: Crizotinib					Reference		
Yang et al. (7) (BRAIN, NCT01724801)	Arm A: Icotinib	0.93 (0.6-1.44), p=0.734	NR	NR	Defined as the time from randomisation to progression of intracranial disease or death from any cause. BMs assessed *via* MRI every 6 weeks according to RECIST v1.1 criteria.	0.56 (0.36-0.90), p=0.014	Time from randomization to increase in symptoms from BMs or any symptoms of deterioration	0.75 (0.44-1.27), p=0.284
	Arm B: WBRT + Platinum-based Chemotherapy	Reference				Reference		Reference
Wu et al. (9) (AURA3, NCT02151981)	Arm A: Osimertinib	NR	NR	NR	Defined as time to intracranial progression or death from any cause. BMs assessed *via* CT or MRI according to RECIST v1.1 criteria.	0.32 (0.15-0.69), p=0.004	NR	NR
	Arm B: Platinum-based Chemotherapy					Reference		
Soria et al. (19) (FLAURA, NCT02296125)	Arm A: Osimertinib	0.83 (0.53-1.30)	Time to disease progression or death from any cause, assessed according to RECIST v.1.1 criteria. Tumors were imaged every 6 weeks until 18 months, then every 12 weeks until disease progression.	0.47 (0.30-0.74), p<0.001	Time to intracranial progression or death from any cause. BMs assessed *via* CT or MRI according to RECIST v1.1 criteria.	0.48 (0.26-0.86), p=0.014	NR	NR
	Arm B: Standard EGFR-TKI (Gefitinib or Erlotinib)	Reference		Reference		Reference		
Novello et al. (21) (ALUR, NCT02604342)	Arm A: Alectinib	NR	Time to disease progression or death from any cause, assessed every 6 weeks *via* CT or MRI using RECIST v1.1 criteria	0.12 (0.05-0.27), p<0.001	NR	NR	Time from randomization to radiographic brain tumour progression on MRI using RECIST criteria	0.16 (0.06-0.43)
	Arm B: Chemotherapy (Pemetrexed OR Docetaxel)			Reference				Reference
Peters et al. (22) (ALEX, NCT02075840)	Arm A: Crizotinib	NR	Time to disease progression or death from any cause. Progression assessed as per RECIST v1.1 criteria.	Reference	NR	NR	Time from randomization to radiographic tumour progression on MRI using RECIST v1.1 criteria. HR is cause-specific HR for CNS progression (excluding pts who had non-CNS progression OR death)	Reference
	Arm B: Alectinib			0.4 (0.25-0.64), p<0.0001				0.18 (0.09-0.36), p<0.0001
Solomon et al. (23–25) (PROFILE 1014, NCT01154140)	Arm A: Crizotinib	1.285 (0.716-2.306), p=0.3991	Time to disease progression or death from any cause. Progression assessed as per RECIST v1.1 criteria.	0.4 (0.23-0.69), p<0.001	NR	NR	Intracranial time to tumor progression was defined as time from randomization to first documentation of objective intracranial progression according to RECIST v1.1 criteria	0.45 (0.19-1.07), p=0.063
	Arm B: Platinum-based Chemotherapy	Reference		Reference				Reference
Wu et al. (26) (PROFILE 1029, NCT01639001)	Arm A: Crizotinib	NR	Time to progression of disease as defined by RECIST v1.1, including primary tumour, or death from any cause. Imaging was done every 6 weeks.	0.497 (0.26-0.95)	NR	NR	The time from randomization to the first objective tumor progression considering only intracranial disease, according to RECIST v1.1 criteria.	0.67 (0.33-1.34), p=0.13
	Arm B: Platinum-based Chemotherapy			Reference				Reference
Zhou et al. (27) (ALESIA, NCT02838420)	Arm A: Alectinib	NR	Time to progression of disease as defined by RECIST v1.1, including primary tumour, or death. Imaging done every 8 weeks.	0.11 (0.05-0.28)	NR	NR	Progression due to newly developed CNS lesions or progression of pre-existing baseline CNS lesions per independent review committee assessment according to RECIST v1.1, imaging done every 8 weeks *via* brain MRI. Competing risk analysis done for HR (cause-specific HR for CNS progression without previous systemic progression reported)	0.14 (0.06-0.3), p<0.0001
	Arm B: Crizotinib			Reference				Reference
Shaw et al. (28) (NCT00932893)	Arm A: Crizotinib	NR	Time to progression of disease as defined by RECIST v1.1, including primary tumour, or death. Imaging done every 6 weeks.	0.67 (0.44-1.03)	NR	NR	NR	NR
	Arm B: Chemotherapy			Reference				
Shaw et al. (29) (ASCEND-5, NCT01828112)	Arm A: Ceritinib	NR	Time to progression of disease as defined by RECIST v1.1, including primary tumour, or death. Imaging done every 6 weeks until l8 months, then every 9 weeks thereafter.	0.5 (0.33-0.76)	NR	NR	NR	NR
	Arm B: Chemotherapy (Pemetrexed or Docetaxel)			Reference				
Soria et al. (20) (ASCEND-4, NCT01828099)	Arm A: Ceritinib	NR	Time to progression of disease as defined by RECIST v1.1, including primary tumour, or death. Imaging done every 6 weeks until 33 months, then every 9 weeks thereafter.	0.7 (0.44-1.12)	NR	NR	NR	NR
	Arm B: Platinum-based Chemotherapy			Reference				
Schuler et al. (30) (LUX-Lung 3, NCT00949650)	Arm A: Afatinib	1.15 (0.49-2.67), p=0.752	Time to progression of disease as defined by RECIST v1.1, including primary tumour, or death. Imaging done every 6 weeks until 4 months, then every 12 weeks until progression.	0.54 (0.12-1.25), p=0.138	NR	NR	NR	NR
	Arm B: Platinum-based Chemotherapy (Cisplatin/Pemetrexed)	Reference		Reference				
Schuler et al. (30) (LUX-Lung 6, NCT01121393)	Arm A: Afatinib	1.13 (0.56-2.26), p=0.732	Time to progression of disease as defined by RECIST v1.1, including primary tumour, or death. Imaging done every 6 weeks until 4 months, then every 12 weeks until progression.	0.47 (0.18-1.21), p=0.106	NR	NR	NR	NR
	Arm B: Platinum-based Chemotherapy (Cisplatin/Gemcitabine)	Reference		Reference				
Park et al. (31) (LUX-Lung 7, NCT01466660.)	Arm A: Afatinib	1.16 (0.61-2.21), p=0.21	Time from randomization to disease progression, pre RECIST v1.1 criteria, or death from any cause. Imaging done every 8 weeks until week 64 then every 12 weeks thereafter.	0.76 (0.41-1.44), p=0.93	NR≈	NR	NR	NR
	Arm B: Gefitinib	Reference		Reference				
Hosomi et al. (32) (NEJ009, UMIN000006340)	Arm A: Gefinitib	Reference	Time from randomization to disease progression, per RECIST v1.1, or death from any cause. Imaging done every 8 weeks until 12 months, then every 12 weeks thereafter	Reference	NR	NR	NR	NR
	Arm B: Gefitinib + Platinum-based Chemotherapy	0.66 (0.4-1.07)		0.32 (0.19-0.53)				
Saito et al. (33) (NEJ026, UMIN000017069)	Arm A: Erlotinib + Bevacizumab	NR	Time from randomization to disease progression as per RECIST v1.1, or death from any cause. Imaging done every 6 weeks until 18 months, then every 12 weeks thereafter.	0.78 (0.42-1.43)	NR	NR	NR	NR
	Arm B: Erlotinib alone			Reference				
Noronha et al. (8) (CTRI/2016/08 /007149)	Arm A: Gefinitib	NR	Time from randomization to disease progression as per RECIST v1.1, or death from any cause. Imaging done every 9 weeks.	Reference	NR	NR	NR	NR
	Arm B: Gefitinib + Platinum-based Chemotherapy			0.53 (0.29-0.98)				

### Efficacy

The efficacy analysis was done using several individual networks, as there was insufficient overlap between all 19 trials to produce a single coherent network graph for each outcome. In addition, not every trial reported all of our outcomes of interest, and analysis of each outcome was done with the available data. Therefore, each efficacy analysis below includes a subset of the nineteen total trials. Supplement E contains league tables showing the results of all pairwise comparisons for each analysis.

### Pooled Analyses of EGFRi or ALKi Versus Conventional Chemotherapy for NSCLC Patients With Brain Metastases

iPFS

**Figure 2:**
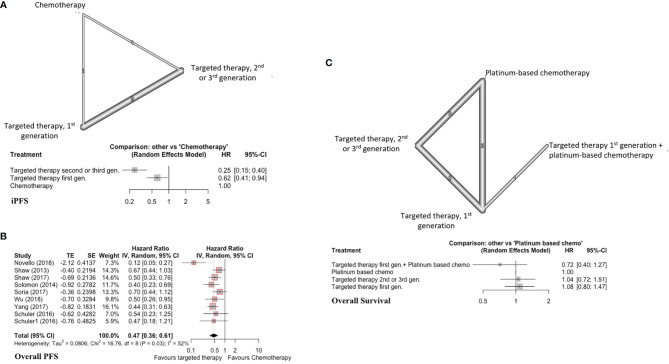
**(A)** iPFS in EGFR-mutated/ALK-rearranged NSCLC. Upper panel - network graph with treatment nodes included in analysis. The number included in the link between treatments indicates the number of studies included in that direct comparison. Lower panel - forest plot showing comparison of included treatment arms in the network meta-analysis, with associated hazard ratios. The treatment with no shown CI was chosen as the reference study arm. **(B)** Forest plot of traditional pairwise meta-analysis comparing all targeted therapies versus traditional chemotherapy for overall PFS in EGFR-mutated/ALK-rearranged NSCLC. **(C)** OS in EGFR-mutated/ALK-rearranged NSCLC, with network graph (upper panel) and forest plot (lower panel).

This analysis included 5 studies, 400 patients with targeted therapy and 114 with conventional chemotherapy. Two focused on patients with ALK re-arrangements and 3 on EGFR mutated patients^7,9(p3),17–19^. We grouped all first-generation targeted therapies together and compared against newer targeted therapies (such as second and third generation). This was done as several individual trials compared first-generation TKIs with second/third generation TKIs, but did not compare different first-generation TKIs against each other. All conventional chemotherapy arms were also grouped together, and we included one study with WBRT added to chemotherapy in the chemotherapy arm (**Figure 2A**) (7). As treatment arms were grouped together, a random effects model was used despite non-significant Q statistic (Q=2.95, df=3, P value=0.39). Both direct and indirect estimates from the model were in agreement (**Supplement C**, **Figure S1**). Targeted therapies were superior to conventional chemotherapy in improving iPFS (**Figure 2A**). Moreover, newer generations TKIs showed greater benefit compared to first generation TKIs (HR=0.39, 95%CI 0.26-0.58), and ranked first in improving iPFS (P-score=1.0) (**Supplement C**, **Figure S2**). The overall certainty of evidence was moderate to high (**Supplement D**, **Table S3**).

#### Overall PFS

Here, we included nine studies with patients harboring either EGFR mutations or ALK rearrangements (n=419 TKI, n=312 conventional chemotherapy) and reporting overall PFS (7, 20, 21, 23, 26, 28–30). This was a traditional pairwise meta-analysis (**Figure 2B**). TKIs significantly improved overall PFS compared to conventional chemotherapy (X^2^ = 16.76, df=8, p=0.03; HR=0.47, 95%CI 0.36-0.61). The overall certainty of evidence was high (**Supplement D**, **Table S4**).

#### Overall Survival

Seven studies were included with 572 total patients (n=376 TKIs, n=146 chemotherapy, n=50 TKI + chemotherapy, with 6 studies focusing on patients with EGFR mutations and one on patients with ALK re-arrangements) (7, 19, 23, 30–32). First generation TKIs were grouped together, and studies combining first generation TKIs with chemotherapy were treated as a separate node. Newer TKIs (second or third generation) were grouped (**Figure 2C**). Both direct and indirect estimates from the model were in agreement (**Supplement C**, **Figure S3**).

Among included treatments, first generation TKI (gefitinib) plus chemotherapy ranked first in improving overall survival (P score=0.91) and showed a trend toward significance (HR=0.72, 95%CI 0.40-1.27) (**Figure 2C**) (**Supplement C**, **Figure S4**). TKIs alone did not improve overall survival compared to platinum-based chemotherapy alone. The overall certainty of evidence was moderate for all comparisons (**Supplement D**, **Table S5**).

### Subgroup Analyses: EGFR Mutant NSCLC With BM

For this set of analyses, we included studies that only enrolled patients with EGFR mutated NSCLC. All first generation EGFRis (gefitinib, erlotinib, icotinib) were grouped.

#### iPFS

**Figure 3:**
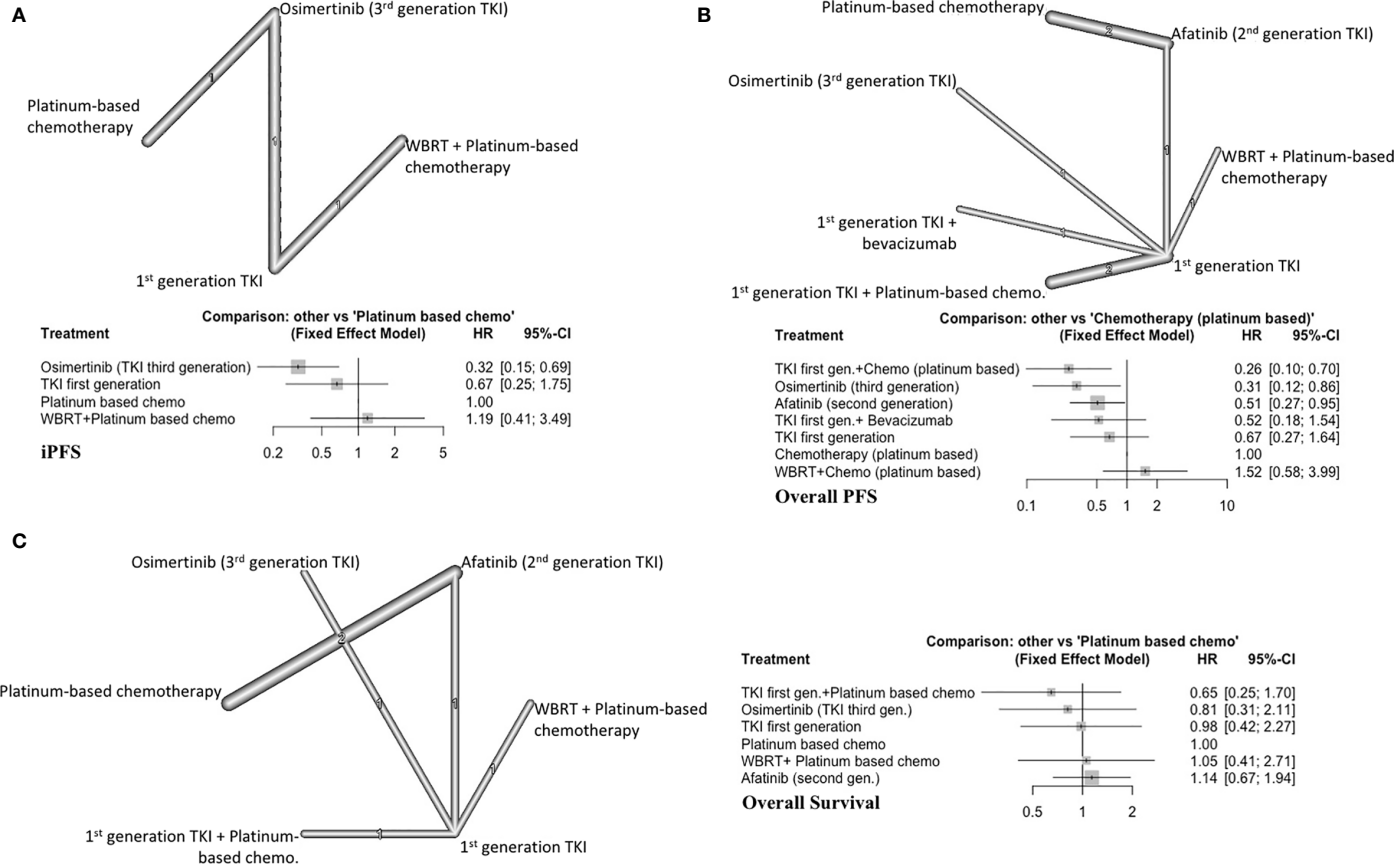
**(A)** iPFS in EGFR-mutated NSCLC, with network graph (upper panel) and forest plot (lower panel), **(B)** Overall PFS in EGFR-mutated NSCLC, with network graph (upper panel) and forest plot (lower panel), **(C)** OS EGFR-mutated NSCLC, with network graph (left panel) and forest plot (right panel).

Three studies with 4 distinct arms of treatment were included in this analysis, with 390 total patients (7, 9, 19). Treatment arms included platinum-based chemotherapy, WBRT plus platinum-based chemotherapy, icotinib (first generation EGFRi), and osimertinib (third generation EGFRi) (**Figure 3A**). Osimertinib significantly improved iPFS (HR=0.32, 95%CI 0.15-0.69) compared to platinum-based chemotherapy alone and ranked first among treatment arms for improving iPFS (P score=0.99) (**Supplement C**, **Figure S5**). Using a first-generation EGFRi or adding WBRT to platinum-based chemotherapy did not improve iPFS (**Figure 3A**). The overall certainty of evidence was low (**Supplement D**, **Table S6**).

#### Overall PFS

Eight different studies were included in this subgroup with 629 total patients (7, 8, 19, 30–33). As a result, seven distinct treatment arms were compared (**Figure 3B**). A fixed effects model was used (Q=1.59, df=2, P value=0.45).

First generation EGFRi (gefitinib) plus platinum-based chemotherapy (P score=0.94) ranked first followed by osimertinib alone (P score=0.84) and afatinib alone (P score=0.57) in improving overall PFS (**Supplement C**, **Figure S6**). WBRT with chemotherapy or first generation EGFRi alone did not improve overall PFS compared to platinum-based chemotherapy alone (**Figure 3B**). Afatinib alone (HR=0.51, 95%CI 0.27-0.95), osimertinib alone (HR=0.31, 95%CI 0.12-0.86) and gefitinib plus platinum-based chemotherapy (HR=0.26, 95%CI 0.10-0.70) improved overall PFS compared to platinum-based chemotherapy alone. The overall certainty of evidence was low (**Supplement D**, **Table S7**).

#### Overall Survival

Six studies were included (493 patients) (7, 19, 30–32). All first-generation EGFRi were grouped together for this analysis, resulting in 6 distinct treatment arms (**Figure 3C**). All the included treatment arms showed similar efficacy as platinum-based chemotherapy and did not significantly increase OS (**Figure 3C**). The overall certainty of evidence was low (**Supplement D**, **Table S8**).

### Subgroup Analyses: ALK Rearranged NSCLC Patients With BM

For these analyses, we compared ALKi with chemotherapy. All conventional chemotherapy arms were entered under the same node (Chemotherapy) in the network.

#### iPFS

**Figure 4:**
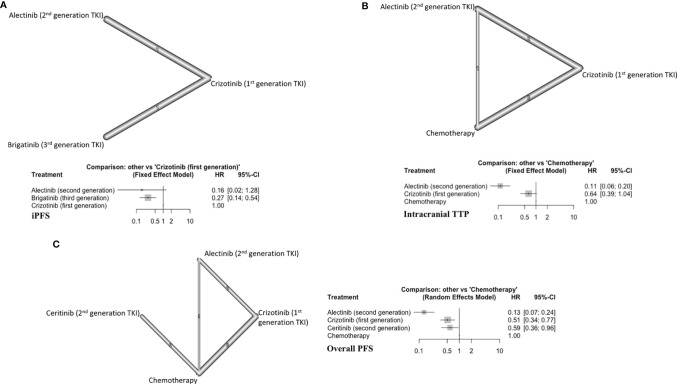
**(A)** iPFS in ALK-rearranged NSCLC, with network (upper panel) and forest plot (lower panel), **(B)** Intracranial TTP in ALK-rearranged NSCLC (upper panel) and forest plot (lower panel), **(C)** Overall PFS in ALK-rearranged NSCLC (left panel) and forest plot (right panel).

Two trials (124 patients) with a total of three arms comparing generations of ALKi were included (**Figure 4A**) (17, 18). Alectinib (second generation TKI) showed a trend toward improving the iPFS (HR=0.16, 95% CI 0.02-1.28) (**Figure 4A**). Alectinib (P score=0.81) ranked first followed by brigatinib (P score =0.65) in improving iPFS (**Supplement C**, **Figure S7**). Brigatinib was superior to crizotinib (first generation ALKi) in prolonging iPFS (HR=0.27, 95%CI 0.14-0.54). The overall certainty of evidence was low for these comparisons (**Supplement D**, **Table S9**).

#### Intracranial TTP

Five studies were included (394 patients) (21–23, 26, 27, 34). The three treatment arms in this subgroup were alectinib, crizotinib, and chemotherapy (**Figure 4B**). Alectinib ranked first for improving iTTP (P score=1) (**Supplement C**, **Figure S8**). Alectinib significantly improved iTTP compared to both crizotinib (HR=0.17, 95%CI 0.11-0.28) and chemotherapy (HR=0.11, 95%CI 0.06-0.20) (**Figure 4B**). Crizotinib showed a trend toward improved iTTP compared to chemotherapy (HR=0.64, 95%CI 0.39-1.04). The overall certainty of evidence was moderate to high (**Supplement D**, **Table S10**).

#### Overall PFS

Eight different studies were included (754 patients) (20–23, 26–29). There were four distinct treatment arms in this analysis (**Figure 4C**). All three targeted therapies improved overall PFS compared to conventional chemotherapy. Alectinib ranked first in improving overall PFS (P score=1) (**Supplement C**, **Figure S9**). The overall certainty of evidence was moderate to high (**Supplement C**, **Table S11**).

### Quality Assessment

The quality assessment of included studies showed an overall low risk of bias in 13/19 trials and 6 trials with ‘some concerns’ overall. There were no studies with an overall high risk of bias. **Supplement B**, **Table S1** shows full RoB 2.0 results for all included studies.

### Adverse Events

All studies reported adverse events, with traditional chemotherapy having similar incidence of grade 3/4 AEs across studies, and most targeted therapies with a similar safety profile. In studies directly comparing any EGFRi alone with EGFRi plus chemotherapy or chemotherapy alone, the EGFRi therapies had a lower incidence of Grade 3/4 AEs (7–9, 30, 32, 33). Among ALKi, alectinib showed a lower incidence of Grade 3/4 AEs than both chemotherapy and crizotinib in direct comparisons (18, 21, 22, 27). **Supplement B**, **Table S2** summarizes the incidence of grade 3/4 AEs across studies.

## Discussion

In this systematic review and NMA, we provide a quantitative comparison showing the superiority of TKIs against conventional chemotherapeutic agents in improving both iPFS and overall PFS in patients with NSCLC with BMs, with a moderate to high degree of certainty. This benefit was greater with newer generations of TKIs. The iPFS/overall PFS benefit with TKIs did not translate to a difference in OS compared to conventional chemotherapy, with or without WBRT. To the best of our knowledge, this is the first study to provide a comprehensive quantitative comparison based on RCT data of the efficacy of TKIs in patients with BMs from NSCLC and activating EGFR mutations or ALK rearrangements, which is an important subpopulation of patients with NSCLC. The use of a NMA allowed for comparisons between treatment arms that have never been directly assessed in existing trials, providing new quantitative insight into the comparative efficacy of these treatments, in addition to the already well-established qualitative superiority of these agents. Previous meta-analyses have demonstrated the efficacy of adding TKI therapy to traditional radiotherapy or chemotherapy approaches in EGFR-mutant patients, similar to our results in this analysis (35–38). However, a recent meta-analysis by Singh et al. found no PFS or OS benefit on addition of TKIs to RT in EGFR or ALK mutant patients (39). Importantly, this study and other past works have included numerous retrospective and non-randomized studies in their analysis, limiting the quality of evidence in each individual analysis. Our work differs from past meta-analyses in that it is the first comprehensive analysis based entirely on RCT data, thereby providing the highest level of evidence to inform future clinical decision-making in this population of patients. Our findings are also in keeping with the National Comprehensive Cancer Network Clinical Practice Guidelines in NSCLC, which recommend first-line TKIs in patients with metastatic disease and activating EGFR or ALK mutations (40).

The improvement of iPFS we observed with newer generations of TKIs is likely in large part due to their proficiency in crossing the BBB, which not only enables targeting of bulk tumor but also micro-metastases (2, 4, 41–44). The current standard of care in NSCLC treatment in many center worldwide already focuses on use of TKIs rather than traditional chemotherapy wherever possible – however, we show significantly increased benefit with the use of newer generations of TKIs. The CNS penetrance of newer TKIs is particularly relevant as we have seen a recent paradigmatic shift in favor of SRS instead of WBRT in the local management of oligometastatic brain disease; while SRS is associated with a lower rate of long-term cognitive decline, the rate of distant BM recurrence is higher than with WBRT (45). Therefore, the use of CNS-penetrating TKIs may help reduce BM recurrence in patients receiving SRS instead of WBRT, or potentially allow select groups of patients to avoid these local treatments altogether. We were unable to find direct comparisons between SRS and TKIs, and indirect comparisons were not feasible. Assessing the efficacy of combinations of SRS and TKI as well as direct head-to-head comparisons of non-inferiority are important areas of future research.

The addition of WBRT to conventional chemotherapy did not improve overall PFS or OS in patients with EGFR mutated NSCLC with BMs. This reaffirms the notion that patients often succumb to their systemic disease and emphasizes the importance of cognitive preservation for as long as possible. Importantly, however, the lack of OS benefit with TKIs despite their intracranial efficacy may be partially explained by patient crossover to TKIs in individual trials after progression on ineffective chemotherapy, which may have confounded the results. This issue was observed in our analysis of overall PFS as well: gefitinib and chemotherapy led to an improvement of overall PFS compared to osimertinib, despite the latter having greater intracranial efficacy. This observation may be related to osimertinib being evaluated as a second-line agent whereas gefitinib and chemotherapy were studied as first-line therapy. Patients with BMs also represent those with more advanced disease, and may therefore be more likely to succumb to their disease independent of treatment. In addition, the combination of EGFR and ALK-positive patients in our analysis may have impacted OS results, since the prognosis of patients with these two activating mutations can differ significantly (23, 46–49).

### Limitations

Using an NMA, we were able to compare the efficacy of different modalities of treatment, specifically, different generations of targeted therapies and conventional chemotherapy against each other in NSCLC with BMs. Conducting numerous RCTs to individually compare each of these treatment options is costly, not feasible, and in some cases unethical. To lower the internal bias, we only included RCTs. As a result, we did not include some other targetable genetic alterations in NSCLC such as *ROS1* translocations, *MET* exon-14-skipping mutations, or *RET* fusions. Further, we were unable to create a single network for each outcome due to several broken links between our included studies and limited outcome data. Therefore, our analysis was completed using several fragmented networks with a subset of studies in each network, limiting the power of each individual analysis. We also combined several treatment arms in order to obtain more robust comparisons; we grouped different generations of TKIs when possible and treated conventional chemotherapy as a single node wherever necessary. Any heterogeneity present within these individual classes may represent a source of confounding, as different chemotherapy regimens and TKIs may have varying efficacy. However, as shown in **Table 1**, the vast majority of the interventions classed as “traditional chemotherapy” used platinum-based doublet regimens or single-agent regimens with pemetrexed or docetaxel, which have been shown to have relatively comparable efficacy in the existing literature (15, 16, 50). In addition, the goal of our work was to perform a high-level class-based analysis of traditional chemotherapy approaches versus newer TKIs in BM patients with NSCLC. Combining classes of similar therapies is necessary to answer this specific question, despite differences in intra-class efficacy that may exist.

We also included several phase 2 trials, which might be at risk of small study bias (28, 31). Our analysis is also limited by the moderate or low certainty of evidence in some cases. Since many of our included studies excluded patients who had symptomatic or otherwise unstable BMs, the results of this work may also not be generalizable to patients suffering acute neurological decline from their BMs. Moreover, we included several studies that only enrolled patients who failed prior TKI or chemotherapy treatment; these patients may be distinct from chemotherapy-naïve patients and might have affected the result (20, 28, 29). Nonetheless, the inclusion of these patients reflects the real-world relevance of our results, as patients seen in everyday practice may often have had several rounds of therapy and stabilizing treatment prior to being considered for successive generations of targeted therapy.

Our study provides a comprehensive analysis of how the various interventions for NSCLC BMs with EGFR mutations/ALK rearrangements rank quantitatively in as close to a “real-world” setting as possible. Furthermore, although the cost-effectiveness of upfront next generation sequencing for known NSCLC mutations has been demonstrated, the cost-effectiveness of the respective generations of TKIs have been limited (51, 52). Our results provide valuable quantitative data on the comparative efficacy of TKIs in comparison to each other and chemotherapy, providing a basis for future work including cost-effectiveness analyses and RCTs focusing on BM patients in NSCLC.

## Conclusions and Implications for Practice

In this work, we conducted a comprehensive systematic review and NMA on patients with either EGFR mutated or ALK rearranged NSCLC with BMs. TKIs showed improved intracranial and overall PFS compared to conventional modalities such as chemotherapy and WBRT, with greater benefit seen using newer generations of TKIs. The incidence of serious adverse events was also lower with most TKIs. Taken together, these results underscore the importance of genetic testing in defining targetable mutations in BMs from NSCLC, support the use of newer generations of TKIs, and point towards the need for the development of further precision therapies for the treatment of this set of tumours. We provide a quantitative basis for the design of future clinical trials evaluating the efficacy of these regimens on the specific cohort of BM patients with NSCLC. Further trials are necessary to establish the efficacy of these treatments in combination with other emerging agents and treatment approaches such as immunotherapy, surgery, and/or radiotherapy, thereby providing more definitive evidence for the management of BMs from NSCLC.

## Data Availability Statement

The original contributions presented in the study are included in the article/**Supplementary Material**. Further inquiries can be directed to the corresponding author.

## Author Contributions

KB, ST, and AM developed the research question. JD, WH, YE, and KB completed data extraction and screening. KB, ST, and AM completed data analysis and wrote the manuscript. All authors contributed to the restructuring and editing of the manuscript. All authors contributed to the article and approved the submitted version.

## Conflict of Interest

The authors declare that the research was conducted in the absence of any commercial or financial relationships that could be construed as a potential conflict of interest.

## Publisher’s Note

All claims expressed in this article are solely those of the authors and do not necessarily represent those of their affiliated organizations, or those of the publisher, the editors and the reviewers. Any product that may be evaluated in this article, or claim that may be made by its manufacturer, is not guaranteed or endorsed by the publisher.

## References

[1] Owen S Souhami L . The Management of Brain Metastases in Non-Small Cell Lung Cancer. Front Oncol (2014) 4:248. doi: 10.3389/fonc.2014.00248 25309873 PMC4164096

[2] Ahluwalia MS Becker K Levy BP . Epidermal Growth Factor Receptor Tyrosine Kinase Inhibitors for Central Nervous System Metastases From Non-Small Cell Lung Cancer. Oncologist (2018) 23(10):1199–209. doi: 10.1634/theoncologist.2017-0572 PMC6263119 29650684

[3] Azzam GA Mellon EA Samuels SE Yechieli RL . The Changing Paradigm of Treatment for Non-Small Cell Lung Cancer Intracranial Metastases. Curr Pulmonol Rep (2018) 7(4):203–13. doi: 10.1007/s13665-018-0215-2

[4] Bartolotti M Franceschi E Brandes AA . EGF Receptor Tyrosine Kinase Inhibitors in the Treatment of Brain Metastases From non-Small-Cell Lung Cancer. Expert Rev Anticancer Ther (2012) 12(11):1429–35. doi: 10.1586/era.12.121 23249107

[5] Andrews DW Scott CB Sperduto PW Flanders AE Gaspar LE Schell MC . Whole Brain Radiation Therapy With or Without Stereotactic Radiosurgery Boost for Patients With One to Three Brain Metastases: Phase III Results of the RTOG 9508 Randomised Trial. Lancet (London England) (2004) 363(9422):1665–72. doi: 10.1016/S0140-6736(04)16250-8 15158627

[6] Lee JS Hong JH Sun DS Won HS Kim YH Ahn MS . The Impact of Systemic Treatment on Brain Metastasis in Patients With Non-Small-Cell Lung Cancer: A Retrospective Nationwide Population-Based Cohort Study. Sci Rep (2019) 9(1):18689. doi: 10.1038/s41598-019-55150-6 31822734 PMC6904708

[7] Yang J-J Zhou C Huang Y Feng J Lu S Song Y . Icotinib Versus Whole-Brain Irradiation in Patients With EGFR-Mutant Non-Small-Cell Lung Cancer and Multiple Brain Metastases (BRAIN): A Multicentre, Phase 3, Open-Label, Parallel, Randomised Controlled Trial. Lancet Respir Med (2017) 5(9):707–16. doi: 10.1016/S2213-2600(17)30262-X 28734822

[8] Noronha V Patil VM Joshi A Menon N Chougule A Mahajan A . Gefitinib Versus Gefitinib Plus Pemetrexed and Carboplatin Chemotherapy in EGFR-Mutated Lung Cancer. JCO (2019) 38(2):124–36. doi: 10.1200/JCO.19.01154 31411950

[9] Wu Y-L Ahn M-J Garassino MC Han J-Y Katakami N Kim HR . CNS Efficacy of Osimertinib in Patients With T790M-Positive Advanced Non-Small-Cell Lung Cancer: Data From a Randomized Phase III Trial (Aura3). J Clin Oncol (2018) 36(26):2702–9. doi: 10.1200/JCO.2018.77.9363 30059262

[10] Rouse B Chaimani A Li T . Network Meta-Analysis: An Introduction for Clinicians. Intern Emerg Med (2017) 12(1):103–11. doi: 10.1007/s11739-016-1583-7 PMC5247317 27913917

[11] RoB 2: A Revised Cochrane Risk-of-Bias Tool for Randomized Trials. Available at: https://methods.cochrane.org/bias/resources/rob-2-revised-cochrane-risk-bias-tool-randomized-trials (Accessed August 2, 2020). 10.1016/j.jclinepi.2020.06.015 32562833

[12] Guyatt GH Oxman AD Vist GE Kunz R Falck-Ytter Y Alonso-Coello P . GRADE: An Emerging Consensus on Rating Quality of Evidence and Strength of Recommendations. BMJ (2008) 336(7650):924–6. doi: 10.1136/bmj.39489.470347.AD PMC2335261 18436948

[13] Nikolakopoulou A Higgins JPT Papakonstantinou T Chaimani A Del Giovane C Egger M . CINeMA: An Approach for Assessing Confidence in the Results of a Network Meta-Analysis. PloS Med (2020) 17(4). doi: 10.1371/journal.pmed.1003082 PMC7122720 32243458

[14] Rücker G Schwarzer G . Ranking Treatments in Frequentist Network Meta-Analysis Works Without Resampling Methods. BMC Med Res Methodol (2015) 15(1):58. doi: 10.1186/s12874-015-0060-8 26227148 PMC4521472

[15] Okamoto I Nokihara H Nomura S Niho S Sugawara S Horinouchi H . Comparison of Carboplatin Plus Pemetrexed Followed by Maintenance Pemetrexed With Docetaxel Monotherapy in Elderly Patients With Advanced Nonsquamous Non–Small Cell Lung Cancer: A Phase 3 Randomized Clinical Trial. JAMA Oncol (2020) 6(5):e196828–e196828. doi: 10.1001/jamaoncol.2019.6828 32163097 PMC7068674

[16] Georgoulias V Papadakis E Alexopoulos A Tsiafaki X Rapti A Veslemes M . Platinum-Based and Non-Platinum-Based Chemotherapy in Advanced Non-Small-Cell Lung Cancer: A Randomised Multicentre Trial. Lancet (2001) 357(9267):1478–84. doi: 10.1016/S0140-6736(00)04644-4 11377599

[17] Camidge DR Kim HR Ahn M-J Yang JC-H Han J-Y Lee J-S . Brigatinib Versus Crizotinib in ALK-Positive Non–Small-Cell Lung Cancer. N Engl J Med (2018) 379(21):2027–39. doi: 10.1056/NEJMoa1810171 30280657

[18] Hida T Nokihara H Kondo M Kim YH Azuma K Seto T . Alectinib Versus Crizotinib in Patients With ALK-Positive Non-Small-Cell Lung Cancer (J-ALEX): An Open-Label, Randomised Phase 3 Trial. Lancet (2017) 390(10089):29–39. doi: 10.1016/S0140-6736(17)30565-2 28501140

[19] Soria J-C Ohe Y Vansteenkiste J Reungwetwattana T Chewaskulyong B Lee KH . Osimertinib in Untreated EGFR-Mutated Advanced Non–Small-Cell Lung Cancer. N Engl J Med (2018) 378(2):113–25. doi: 10.1056/NEJMoa1713137 29151359

[20] Soria J-C Tan DSW Chiari R Wu Y-L Paz-Ares L Wolf J . First-Line Ceritinib Versus Platinum-Based Chemotherapy in Advanced ALK-Rearranged Non-Small-Cell Lung Cancer (ASCEND-4): A Randomised, Open-Label, Phase 3 Study. Lancet (2017) 389(10072):917–29. doi: 10.1016/S0140-6736(17)30123-X 28126333

[21] Novello S Mazières J Oh I-J de Castro J Migliorino MR Helland Å . Alectinib Versus Chemotherapy in Crizotinib-Pretreated Anaplastic Lymphoma Kinase (ALK)-Positive Non-Small-Cell Lung Cancer: Results From the Phase III ALUR Study. Ann Oncol (2018) 29(6):1409–16. doi: 10.1093/annonc/mdy121 PMC6005013 29668860

[22] Peters S Camidge DR Shaw AT Gadgeel S Ahn JS Kim D-W . Alectinib Versus Crizotinib in Untreated ALK-Positive Non–Small-Cell Lung Cancer. N Engl J Med (2017) 377(9):829–38. doi: 10.1056/NEJMoa1704795 28586279

[23] Solomon BJ Mok T Kim D-W Wu Y-L Nakagawa K Mekhail T . First-Line Crizotinib Versus Chemotherapy in ALK-Positive Lung Cancer. N Engl J Med (2014) 371(23):2167–77. doi: 10.1056/NEJMoa1408440 25470694

[24] Solomon BJ Cappuzzo F Felip E Blackhall FH Costa DB Kim D-W . Intracranial Efficacy of Crizotinib Versus Chemotherapy in Patients With Advanced ALK-Positive Non-Small-Cell Lung Cancer: Results From PROFILE 1014. J Clin Oncol (2016) 34(24):2858–65. doi: 10.1200/JCO.2015.63.5888 27022118

[25] Solomon BJ Kim D-W Wu Y-L Nakagawa K Mekhail T Felip E . Final Overall Survival Analysis From a Study Comparing First-Line Crizotinib Versus Chemotherapy in ALK-Mutation-Positive Non-Small-Cell Lung Cancer. J Clin Oncol (2018) 36(22):2251–8. doi: 10.1200/JCO.2017.77.4794 29768118

[26] Wu Y-L Lu S Lu Y Zhou J Shi Y Sriuranpong V . Results of PROFILE 1029, a Phase III Comparison of First-Line Crizotinib Versus Chemotherapy in East Asian Patients With ALK-Positive Advanced Non–Small Cell Lung Cancer. J Thorac Oncol (2018) 13(10):1539–48. doi: 10.1016/j.jtho.2018.06.012 29966800

[27] Zhou C Kim S-W Reungwetwattana T Zhou J Zhang Y He J . Alectinib Versus Crizotinib in Untreated Asian Patients With Anaplastic Lymphoma Kinase-Positive Non-Small-Cell Lung Cancer (ALESIA): A Randomised Phase 3 Study. Lancet Respir Med (2019) 7(5):437–46. doi: 10.1016/S2213-2600(19)30053-0 30981696

[28] Shaw AT Kim D-W Nakagawa K Seto T Crinó L Ahn M-J . Crizotinib Versus Chemotherapy in Advanced ALK-Positive Lung Cancer. N Engl J Med (2013) 368(25):2385–94. doi: 10.1056/NEJMoa1214886 23724913

[29] Shaw AT Kim TM Crinò L Gridelli C Kiura K Liu G . Ceritinib Versus Chemotherapy in Patients With ALK-Rearranged Non-Small-Cell Lung Cancer Previously Given Chemotherapy and Crizotinib (ASCEND-5): A Randomised, Controlled, Open-Label, Phase 3 Trial. Lancet Oncol (2017) 18(7):874–86. doi: 10.1016/S1470-2045(17)30339-X 28602779

[30] Schuler M Wu Y-L Hirsh V O'Byrne K Yamamoto N Mok T . First-Line Afatinib Versus Chemotherapy in Patients With Non–Small Cell Lung Cancer and Common Epidermal Growth Factor Receptor Gene Mutations and Brain Metastases. J Thorac Oncol (2016) 11(3):380–90. doi: 10.1016/j.jtho.2015.11.014 26823294

[31] Park K Tan E-H O’Byrne K Zhang L Boyer M Mok T . Afatinib Versus Gefitinib as First-Line Treatment of Patients With EGFR Mutation-Positive Non-Small-Cell Lung Cancer (LUX-Lung 7): A Phase 2B, Open-Label, Randomised Controlled Trial. Lancet Oncol (2016) 17(5):577–89. doi: 10.1016/S1470-2045(16)30033-X 27083334

[32] Hosomi Y Morita S Sugawara S Kato T Fukuhara T Gemma A . Gefitinib Alone Versus Gefitinib Plus Chemotherapy for Non-Small-Cell Lung Cancer With Mutated Epidermal Growth Factor Receptor: NEJ009 Study. J Clin Oncol (2020) 38(2):115–23. doi: 10.1200/JCO.19.01488 31682542

[33] Saito H Fukuhara T Furuya N Watanabe K Sugawara S Iwasawa S . Erlotinib Plus Bevacizumab Versus Erlotinib Alone in Patients With EGFR-Positive Advanced Non-Squamous Non-Small-Cell Lung Cancer (NEJ026): Interim Analysis of an Open-Label, Randomised, Multicentre, Phase 3 Trial. Lancet Oncol (2019) 20(5):625–35. doi: 10.1016/S1470-2045(19)30035-X 30975627

[34] Gadgeel S Peters S Mok T Shaw AT Kim DW Ou SI . Alectinib Versus Crizotinib in Treatment-Naive Anaplastic Lymphoma Kinase-Positive (ALK Plus) Non-Small-Cell Lung Cancer: CNS Efficacy Results From the ALEX Study. Ann Oncol (2018) 29(11):2214–22. doi: 10.1093/annonc/mdy405 PMC6290889 30215676

[35] Jiang T Min W Li Y Yue Z Wu C Zhou C . Radiotherapy Plus EGFR TKIs in Non-Small Cell Lung Cancer Patients With Brain Metastases: An Update Meta-Analysis. Cancer Med (2016) 5(6):1055–65. doi: 10.1002/cam4.673 PMC4924363 26990668

[36] Soon YY Leong CN Koh WY Tham IWK . EGFR Tyrosine Kinase Inhibitors Versus Cranial Radiation Therapy for EGFR Mutant Non-Small Cell Lung Cancer With Brain Metastases: A Systematic Review and Meta-Analysis. Radiother Oncol (2015) 114(2):167–72. doi: 10.1016/j.radonc.2014.12.011 25583566

[37] Du X-J Pan S-M Lai S-Z Xu X-N Deng M-L Wang X-H . Upfront Cranial Radiotherapy vs. EGFR Tyrosine Kinase Inhibitors Alone for the Treatment of Brain Metastases From Non-Small-Cell Lung Cancer: A Meta-Analysis of 1465 Patients. Front Oncol (2018) 8:603. doi: 10.3389/fonc.2018.00603 30619745 PMC6299879

[38] Zheng H Liu Q-X Hou B Zhou D Li J-M Lu X . Clinical Outcomes of WBRT Plus EGFR-TKIs Versus WBRT or TKIs Alone for the Treatment of Cerebral Metastatic NSCLC Patients: A Meta-Analysis. Oncotarget (2017) 8(34):57356–64. doi: 10.18632/oncotarget.19054 PMC5593647 28915676

[39] Singh R Lehrer EJ Ko S Peterson J Lou Y Porter AB . Brain Metastases From Non-Small Cell Lung Cancer With EGFR or ALK Mutations: A Systematic Review and Meta-Analysis of Multidisciplinary Approaches. Radiother Oncol (2020) 144:165–79. doi: 10.1016/j.radonc.2019.11.010 31812932

[40] National Comprehensive Cancer Network Guidelines . Non-Small Cell Lung Cancer, Version 7.2021 (2021). Available at: https://www.nccn.org/guidelines/guidelines-detail?category=1&id=1450.

[41] Pardridge WM . Drug Transport Across the Blood-Brain Barrier. J Cereb Blood Flow Metab (2012) 32(11):1959–72. doi: 10.1038/jcbfm.2012.126 PMC3494002 22929442

[42] Sun YW Xu J Zhou J Liu WJ . Targeted Drugs for Systemic Therapy of Lung Cancer With Brain Metastases. Oncotarget (2017) 9(4):5459–72. doi: 10.18632/oncotarget.23616 PMC5797064 29435193

[43] Liam CK . Central Nervous System Activity of First-Line Osimertinib in Epidermal Growth Factor Receptor-Mutant Advanced Non-Small Cell Lung Cancer. Ann Transl Med (2019) 7(3):61. doi: 10.21037/atm.2018.12.68 30906765 PMC6389567

[44] Yang JC-H Cho BC Kim D-W Kim S-W Lee J-S Su W-C . Osimertinib for Patients (Pts) With Leptomeningeal Metastases (LM) From EGFR-Mutant Non-Small Cell Lung Cancer (NSCLC): Updated Results From the BLOOM Study. JCO (2017) 35(15_suppl):2020–0. doi: 10.1200/JCO.2017.35.15_suppl.2020

[45] Aoyama H Shirato H Tago M Nakagawa K Toyoda T Hatano K . Stereotactic Radiosurgery Plus Whole-Brain Radiation Therapy vs Stereotactiv Radiosurgery Alone for Treatment of Brain Metastases. J Am Med Assoc (2006) 295(21):2483–90. doi: 10.1001/jama.295.21.2483 16757720

[46] Kim H Lee H Hong H Kim YJ Kim KG Jeon YK . The Prognostic Implications of EGFR Mutation and ALK Rearrangement for the Long-Term Outcomes of Patients With Resected Lung Adenocarcinomas. Thorac Cancer (2019) 10(7):1619–27. doi: 10.1111/1759-7714.13128 PMC6610284 31215177

[47] Li P Gao Q Jiang X Zhan Z Yan Q Li Z . Comparison of Clinicopathological Features and Prognosis Between ALK Rearrangements and EGFR Mutations in Surgically Resected Early-Stage Lung Adenocarcinoma. J Cancer (2019) 10(1):61–71. doi: 10.7150/jca.26947 30662526 PMC6329857

[48] Nakamura M Kageyama S Niho S Okumura M Hojo H Motegi A . Impact of EGFR Mutation and ALK Translocation on Recurrence Pattern After Definitive Chemoradiotherapy for Inoperable Stage III Non-Squamous Non–small-Cell Lung Cancer. Clin Lung Cancer (2019) 20(3):e256–64. doi: 10.1016/j.cllc.2019.02.021 30926356

[49] Kuan F-C Kuo L-T Chen M-C Yang C-T Shi C-S Teng D . Overall Survival Benefits of First-Line EGFR Tyrosine Kinase Inhibitors in EGFR-Mutated Non-Small-Cell Lung Cancers: A Systematic Review and Meta-Analysis. Br J Cancer (2015) 113(10):1519–28. doi: 10.1038/bjc.2015.356 PMC4815883 26461059

[50] Amarasena IU Chatterjee S Walters JAE Wood-Baker R Fong KM . Platinum Versus Non-Platinum Chemotherapy Regimens for Small Cell Lung Cancer. Cochrane Database Syst Rev (2015) 8:CD006849. doi: 10.1002/14651858.CD006849.pub3 PMC7263420 26233609

[51] Aguiar PN Haaland B Park W San Tan P del Giglio A de Lima Lopes G . Cost-Effectiveness of Osimertinib in the First-Line Treatment of Patients With EGFR-Mutated Advanced Non–Small Cell Lung Cancer. JAMA Oncol (2018) 4(8):1080–4. doi: 10.1001/jamaoncol.2018.1395 PMC6143050 29852038

[52] Pennell NA Mutebi A Zhou ZY Ricculli ML Tang W Wang H . Economic Impact of Next-Generation Sequencing Versus Single-Gene Testing to Detect Genomic Alterations in Metastatic Non–Small-Cell Lung Cancer Using a Decision Analytic Model. JCO Precis Oncol (2019) 3):1–9. doi: 10.1200/PO.18.00356 35100695

