# Supplementary material for: Comparative Efficacy of Systemic Agents for Brain Metastases From Non-Small-Cell Lung Cancer With an EGFR Mutation/ALK Rearrangement: A Systematic Review and Network Meta-Analysis
Source: Front Oncol. 2021 Dec 7;11:739765. doi: 10.3389/fonc.2021.739765 (PMC8691653; doi:10.3389/fonc.2021.739765)
Supplement: Supplementary file 1 [file DataSheet_1.docx]

**Supplement A**

Search strategies for each database are provided below.

CENTRAL:

#1 (brain metastas* OR metastasis to the brain OR secondary brain neoplasm OR secondary brain cancer OR metastatic brain cancer OR metastatic brain disease OR cancer metastasis brain OR metastatic tumor brain OR secondary brain tumor OR metastasis temporal lobe OR metastasis cerebellar):ti,ab,kw in Trials 2099

#2 MeSH descriptor: [Brain Neoplasms] explode all trees 1745

#3 MeSH descriptor: [Brain] explode all trees 11059

#4 #2 OR #3 in Trials 12534

#5 MeSH descriptor: [Neoplasm Metastasis] explode all trees 4573

#6 #4 AND #5 99

#7 MeSH descriptor: [Brain Neoplasms] explode all trees and with qualifier(s): [secondary - SC] 301

#8 #1 OR #6 OR #7 2125

#9 MeSH descriptor: [Radiotherapy] explode all trees 5746

#10 (radiation therap* OR radiotherap* OR targeted radiation therap* OR targeted radiotherap*):ti,ab,kw 28752

#11 #9 OR #10 29105

#12 (gamma knife OR radiosurg* OR stereotactic radiosurg* OR cyberknife radiosurg* OR stereotactic radiation):ti,ab,kw 1050

#13 MeSH descriptor: [Drug Therapy] explode all trees 132242

#14 (chemotherap* OR drug therap* OR pharmacotherap* OR therapy, drug OR antineoplastic OR pharmacologic therapy OR drug treatment):ti,ab,kw 418579

#15 (molecular NEAR/3 therap* NEAR/3 targeted):ti,ab,kw 195

#16 #13 OR #14 OR #15 445062

#17 MeSH descriptor: [Immunotherapy] explode all trees 7464

#18 (immunotherap* OR immunological therapy OR adoptive immunotherap* OR cellular therap*):ti,ab,kw 13257

#19 #17 or #18 18518

#20 (complete resection OR resection OR surgical treatment OR surgical resection OR surgery):ti,ab,kw 165470

#21 #11 AND (#12 OR #16 OR #19 OR #20) 20600

#22 #12 AND (#16 OR #19 OR #20) 730

#23 #16 AND (#19 OR #20) 66948

#24 #19 AND #20 1744

#25 #8 AND (#21 OR #22 OR #23 OR #24) in Trials 933

Embase:

1 exp brain metastasis/ (27564)

2 (brain metastas* or metastasis to the brain or secondary brain neoplasm or secondary brain cancer or metastatic brain cancer or metastatic brain disease or cancer metastasis brain or metastatic tumour brain or secondary brain tumor or metastasis temporal lobe or metastasis cerebellar).mp. (31057)

3 ((brain or cerebellum or cerebellar or temporal) adj4 metastas*).mp. (32832)

4 1 or 2 or 3 (32920)

5 exp radiotherapy/ (533864)

6 (radiation therap* or radiotherap* or targeted radiation therap* or targeted radiotherap*).mp. (574771)

7 5 or 6 (696887)

8 exp radiosurgery/ (60626)

9 (gamma knife or radiosurg* or stereotactic radiosurg* or cyberknife radiosurg* or stereotactic radiation).mp. (27856)

10 8 or 9 (64755)

11 exp surgery/ (4749424)

12 (complete resection or resection or surgical treatment or surgical resection or surgery).mp. (3577150)

13 11 or 12 (5506047)

14 exp "molecularly targeted therapy"/ (25649)

15 (molecular adj4 therap* adj4 targeted).mp. (5720)

16 14 or 15 (28957)

17 exp drug therapy/ (2536721)

18 (chemotherap* or drug therap* or pharmacotherap* or therapy, drug or antineoplastic or pharmacologic therapy or drug treatment).mp. (4736423)

19 17 or 18 (5400096)

20 exp immunotherapy/ (185570)

21 (immunotherap* or immunological therapy or adoptive immunotherap* or cellular therap*).mp. (171919)

22 20 or 21 (221495)

23 7 and (10 or 13 or 16 or 19 or 22) (479380)

24 10 and (13 or 16 or 19 or 22) (63768)

25 13 and (16 or 19 or 22) (1289067)

26 16 and (19 or 22) (20632)

27 19 and 22 (113843)

28 4 and (23 or 24 or 25 or 26 or 27) (17303)

29 ('crossover procedure' or 'double-blind procedure' or 'randomized controlled trial' or 'single-blind procedure').de. or (random* or factorial* or crossover* or (cross adj1 over*) or placebo* or (doubl* adj1 blind*) or (singl* adj1 blind*) or assign* or allocat* or volunteer*).de,ab,ti. (2318175)

30 28 and 29 (1902)

31 (retrospective or systematic review or review or meta-analysis or case series or case report).ti. (861862)

32 30 not 31 (1768)

33 limit 30 to (clinical trial or randomized controlled trial or controlled clinical trial or multicenter study or phase 1 clinical trial or phase 2 clinical trial or phase 3 clinical trial or phase 4 clinical trial) (744)

MEDLINE:

1 (brain metastas* or metastasis to the brain or secondary brain neoplasm or secondary brain cancer or metastatic brain cancer or metastatic brain disease or cancer metastasis brain or metastatic tumour brain or secondary brain tumor or metastasis temporal lobe or metastasis cerebellar).tw,kf. (11036)

2 ((brain or cerebellum or cerebellar or temporal) adj4 metastas*).tw,kf. (12602)

3 (exp brain neoplasms/ or exp brain/) and exp neoplasm metastasis/ (5833)

4 exp brain neoplasms/sc (13066)

5 1 or 2 or 3 or 4 (22706)

6 exp radiotherapy/ (170784)

7 (radiation therap* or radiotherap* or targeted radiation therap* or targeted radiotherap*).tw,kf. (217428)

8 6 or 7 (299457)

9 (gamma knife or radiosurg* or stereotactic radiosurg* or cyberknife radiosurg* or stereotactic radiation).tw,kf. (13186)

10 exp drug therapy/ (1262700)

11 (chemotherap* or drug therap* or pharmacotherap* or therapy, drug or antineoplastic or pharmacologic therapy or drug treatment).tw,kf. (489392)

12 (molecular adj4 therap* adj4 targeted).tw,kf. (3547)

13 10 or 11 or 12 (1596275)

14 exp immunotherapy/ (254948)

15 (immunotherap* or immunological therapy or adoptive immunotherap* or cellular therap*).tw,kf. (75381)

16 14 or 15 (296952)

17 (complete resection or resection or surgical treatment or surgical resection or surgery).tw,kf. (1351040)

18 8 and (9 or 13 or 16 or 17) (146141)

19 9 and (13 or 16 or 17) (6074)

20 13 and (16 or 17) (179883)

21 16 and 17 (6748)

22 5 and (18 or 19 or 20 or 21) (5938)

23 randomized controlled trial.pt. (469642)

24 controlled clinical trial.pt. (92694)

25 randomized.ab. (423793)

26 placebo.ab. (192399)

27 drug therapy.fs. (2053611)

28 randomly.ab. (298610)

29 trial.ab. (441496)

30 groups.ab. (1841268)

31 23 or 24 or 25 or 26 or 27 or 28 or 29 or 30 (4297002)

32 exp animals/ not humans.sh. (4503952)

33 31 not 32 (3714732)

34 22 and 33 (1983)

35 limit 34 to (adaptive clinical trial or clinical trial, all or clinical trial, phase i or clinical trial, phase ii or clinical trial, phase iii or clinical trial, phase iv or clinical trial or controlled clinical trial or equivalence trial or pragmatic clinical trial or randomized controlled trial) (398)

Web of Science:


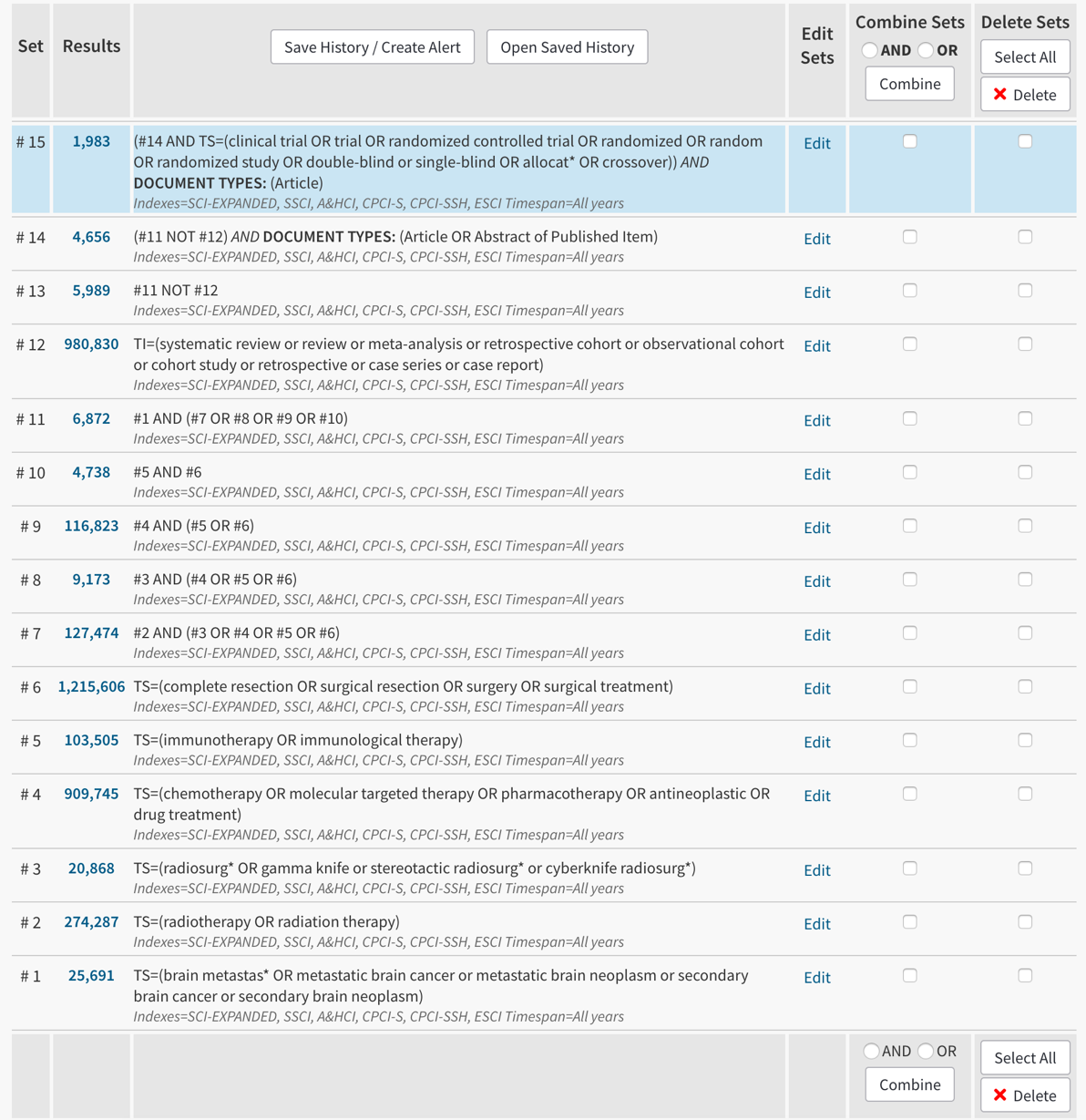


**Supplement B**

Table S1 – RoB 2.0 assessment of included studies

| **Study ID** | **Randomization process** | **Deviations from intended interventions** | **Missing outcome data** | **Measurement of the outcome** | **Selection of the reported result** | **Overall Bias** |
| --- | --- | --- | --- | --- | --- | --- |
| Camidge 2018 | Some concerns | Low | Low | Low | Low | Some concerns |
| Hida 2017 | Low | Low | Low | Low | Low | Low |
| Yang 2017 | Low | Low | Low | Low | Low | Low |
| Wu 2018 (AURA 3) | Low | Low | Low | Low | Low | Low |
| Soria 2018 | Some concerns | Some concerns | Low | Low | Low | Some concerns |
| Novello 2018 | Low | Low | Low | Low | Low | Low |
| Peters 2017 | Low | Low | Low | Low | Low | Low |
| Solomon 2014 | Low | Low | Low | Low | Low | Low |
| Wu 2018 (Profile 1029) | Some concerns | Some concerns | Low | Some concerns | Low | Some concerns |
| Zhou 2019 | Low | Low | Low | Low | Low | Low |
| Shaw 2013 | Low | Low | Low | Low | Low | Low |
| Shaw 2017 | Low | Low | Low | Low | Low | Low |
| Soria 2017 | Low | Low | Low | Low | Low | Low |
| Schuler 2016 (LUX-Lung 3) | Some concerns | Some concerns | Low | Some concerns | Low | Some concerns |
| Schuler 2016 (LUX-Lung 6) | Some concerns | Some concerns | Low | Some concerns | Low | Some concerns |
| Park 2016 | Low | Low | Low | Low | Low | Low |
| Hosomi 2020 | Some concerns | Low | Low | Some concerns | Low | Some concerns |
| Saito 2019 | Low | Low | Low | Low | Low | Low |
| Norohna 2020 | Low | Low | Low | Low | Low | Low |

Table S2 – Grade 3 or 4 adverse events reported in each study

| Study | Events reported for whole-group or BM patients only? | Arm | Description of Adverse Events |
| --- | --- | --- | --- |
| Camidge 2018 | Whole group | Arm A: Brigatinib | Total N in group = 137. **Total Grade ≥ 3 events = 83 (61%).** Major serious AE include increased blood creatine kinase level (22), Hypertension (13), Increased lipase level (18), Increased amylase level (7) |
|  | Whole group | Arm B: Crizotinib | Total N in group = 138. **Total Grade ≥ 3 events = 76 (55%).** Major serious AE include increased alanine aminotransferase level (13), Increased lipase level (7) and Neutropenia (6) |
| Hida 2017 | Whole group | Arm A: Alectinib | Total N in group = 103. **Total Grade 3/4 events = 27 (26%).** Constipation (1), AST increase (1), ALT increase (1), CK increase (5), neutropenia (2), QT prolongation (2), pyrexia (1), maculopapular rash (3), interstitial lung disease (5), decreased appetite (1), anemia (1) |
|  | Whole group | Arm B: Crizotinib | Total N in group = 104. **Total Grade 3/4 events = 54 (52%)**. Nausea (2), diarrhea (2), constipation (1), vomiting (2), esophagitis (2), AST increase (5), ALT increase (13), CK increase (3), neutrophil decrease (14), QT prolongation (7), peripheral edema, (1), rash (1), maculopapular rash (1), interstitial lung disease (3), decreased appetite (1), abnormal hepatic function (6) |
| Yang 2017 | BM patients | Arm A: Icotinib | Total N in group = 85. **Total Grade >3 events = 7 (8%).** Elevated ALT (1), elevated AST (1), impaired hepatic function (1), leucopenia (1), neutropenia (1), dizziness (1), limb numbness (2). |
|  | BM patients | Arm B: WBRT | Total N in group = 73. **Total Grade >3 events = 28 (38%).** Elevated ALT (2), elevated AST (2) impaired hepatic function (5), anemia (8), leucopenia (9), neutropenia (13), elevated ALP (2), hematuria (1), myelosuppression (1), thrombocytopenia (11), myalgia (1), diarrhea (1) |
| Wu 2018 (AURA3) | BM patients | Arm A: Osimertinib | Total N in group = 75. **Total Grade >3 events = 14 (19%).** |
|  | BM patients | Arm B: Platinum-pemetrexed | Total N in group = 41. **Total Grade >3 events = 18 (44%).** |
| Soria 2018 | Whole group | Arm A: Osimertinib | Total N in group **=** 279. **Total Grade ≥ 3 events = 89 (32%).** Major serious AE include Dry skin (6), Decreased appetite (7), Prolonged QT interval on ECG (6) |
|  | Whole group | Arm B: EGFR-TKI (gefitinib or erlotinib) | Total N in group = 277. **Total Grade ≥ 3 events = 114 (41%).** Major serious AE include Rash/acne (19), Diarrhea (6), Decreased appetite (5), Aspartate aminotransferase elevation (12), Alanine aminotransferase elevation (25) |
| Novello 2018 | Whole group | Arm A: Alectinib | Total N in group = 70. **Total Grade >3 events = 19 (27.1%).** Asthenia (2), pneumonia (2), syncope (2), anemia (1), acute kidney injury (2) |
|  | Whole group | Arm B: Chemotherapy | Total N in group = 34. **Total Grade >3 events = 14 (41.2%).** Asthenia (1), fatigue (3), anemia (2), neutropenia (4), febrile neutropenia (2), stomatitis (2). |
| Peters 2017 | Whole group | Arm A: Crizotinib | Total N in group = 151. **Total Grade >3 events = 76 (50%).** Nausea (5), diarrhea (3), vomiting (5), ALT increase (22), AST increase (16), GGT increase (2), peripheral edema (1), anemia (1) |
|  | Whole group | Arm B: Alectinib | Total N in group = 152. **Total Grade >3 events = 63 (41%).** Nausea (1), ALT increase (7), AST increase (8), bilirubin increase (3), weight increase (1), GGT increase (1), anemia (7), photosensitivity reaction (1). |
| Solomon 2014/16/18 | Whole group | Arm A: Crizotinib | Total N in group = 171. **Total Grade >3 events = 98 (57%).** Elevated transaminases (24), decreased appetite (4), diarrhea (4), QT prolongation (4), leukopenia (3), hyponatremia (1), neutropenia (10), pulmonary embolism (11), dyspnea (5), fatigue (5), pneumonia (4), hypophosphatemia (4), vomiting (3), hypokalemia (3); Grade 5: septic shock (2), acute respiratory failure (1), diabetic ketoacidosis (1). |
|  | Whole group | Arm B: Chemotherapy | Total N in group = 169. **Total Grade >3 events = 103 (61%).** Elevated transaminases (4), decreased appetite (1), diarrhea (1), leukopenia (9), hyponatremia (4), anemia (15), thrombocytopenia (11), neutropenia (26), pulmonary embolism (11), dyspnea (4), fatigue (4), pneumonia (2), hypophosphatemia (2), vomiting (5), hypokalemia (4);grade 5: cardiac arrest (1), suicide (1), hemoptysis( |
| Wu 2018 (PROFILE 1029) | Whole group | Arm A: Crizotinib | Total N in group = 104. **Total Grade >3 events (only frequency >15% reported) = 43 (41%).** Increased transaminases (12), edema (1), headache (1), abdominal pain (1), leukopenia (3), neutropenia (17*),* anemia (3), thrombocytopenia (2), hyponatremia (1), cough (1), blood albumin decreased (1). |
|  | Whole group | Arm B: Chemotherapy | Total N in group = 101. **Total Grade >3 events (only frequency >15% reported) = 68 (67%).** Increased transaminases (4), leukopenia (10), neutropenia (25), anemia (13), decreased appetite (1), thrombocytopenia (10), hyponatremia (5). |
| Zhou 2019 | Whole group | Arm A: Alectinib | Total N in group = 125. **Total Grade 3 or 4 events = 36 (29%).** Increased ALT (2), Increased creatine phosphokinase (6), increased bilirubin (1), increased conjugated bilirubin (2), nausea (1), weight gain (4), rash (1), increased GGT (1), blood uric acid increase (3), other (15) |
|  | Whole group | Arm B: Crizotinib | Total N in group = 62. **Total Grade 3 or 4 events = 30 (48%).** Increased ALT (4), increased creatine phosphokinase (2), increased conjugated bilirubin (1), nausea (2), vomiting (2), decreased appetite (3), weight gain (1), rash (2), decreased WBC count (1), headache (1), decreased neutrophil count (7), hyponatremia (3), blood uric acid increase (1) |
| Shaw 2013 | Whole group | Arm A: Crizotinib | Total N in group = 172. **Total Grade 3 or 4 events (only frequency >3% reported) = 94** **(54.6%).** Elevated transaminases (27), PE (9), dyspnea (7), pneumonia (6), hypokalemia (6), QTc prolongation (6), neutropenia (23), anemia (4), WBC decreased (2), fatigue (4. |
|  | Whole group | Arm B: Docetaxel or Pemetrexed | Total N in group = 171. **Total Grade 3 or 4 events (only frequency >3% reported) = 88** **(51.5%).** Elevated transaminases (4), PE (3), dyspnea (5), pneumonia (3), neutropenia (33), anemia (9), WBC decreased (8), fatigue (7). |
| Shaw 2017 | Whole group | Arm A: Ceritinib | Total N in group = 115. **Total Grade 3 or 4 adverse events (frequency >10% reported) = 126.** Diarrhea (5), nausea (9), vomiting (9), ALT increased (24), decreased appetite (2), AST increased (16), weight decreased (3), fatigue (6), asthenia (6), blood ALP increased (7), abdominal pain (1), back pain (1), headache (1), pyrexia (2), upper abdominal pain (1), dyspnea (6), non-cardiac chest pain (1), prolonged QT interval (1), neutropenia (1), GGT increased (24). |
|  | Whole group | Arm B: Chemotherapy (Docetaxel + Pemetrexed) | Total N in group = 113. **Total Grade 3 or 4 adverse events (frequency >10% reported) = 57.** Diarrhea (1), nausea (2), vomiting (1), ALT increase (2), decreased appetite (3), AST increased (1), weight decreased (1), fatigue (5), asthenia (7), abdominal pain (1), back pain (3), headache (2), cough (1), dyspnea (7), arthralgia (3), neutropenia (17), GGT increase (1). |
| Soria 2017 | Whole group | Arm A: Ceritinib | Total N in group = 189. **Total Grade >3 events (only frequency >15% reported) = 148 (78%).** Diarrhea (10), nausea (5), vomiting (10), ALT increase (58), AST increase (32), GGT increase (54), decreased appetite (2), alkaline phosphatase increase (14), fatigue (8), abdominal pain (4), weight decrease (7), creatinine increase (4), upper abdominal pain (3), non-cardiac chest pain (2), back pain (3), asthenia (5), dyspnea (4), anemia (4), neutropenia (1). |
|  | Whole group | Arm B: Chemotherapy | Total N in group = 170. **Total patients with Grade >3 events (only frequency >15% reported) = 108 (62%).** Diarrhea (2), nausea (9), vomiting (10), ALT increase (5), AST increase (3), GGT increase (3), decreased appetite (2), alkaline phosphatase increase (1), fatigue (5), weight decrease (1), non-cardiac chest pain (1), back pain (4), asthenia (6), pyrexia (2), headache (2), dyspnea (11), anemia (13), neutropenia (19), WBC count decreased (7). |
| Schuler 2016 (LUX-Lung 3) | BM patients only | Arm A: Afatinib | Total N in group = 26. **Total patients with Grade >3 events (only frequency >5% reported) = 12 (46%).** Rash (4), diarrhea (3), nail effect (4), stomatitis (2), vomiting (1), hypokalemia (2) |
|  | BM patients only | Arm B: Cisplatin/Pemetrexed | Total N in group = 14. **Total patients with Grade >3 events (only frequency >5% reported) = 8 (57%).** Vomiting (2), nausea (1), fatigue e(1), leukopenia (3), neutropenia (5), atrial fibrillation (1), sodium decrease (1), cardiac failure (1), GGT increased (1), hemoglobin decrease (1), hypokalemia (1), hyponatremia (2), lymphopenia (1), decreased neutrophils (1), pain (1), platelets decreased (1), syncope (1), thrombocytopenia (1) |
| Schuler 2016 (LUX-Lung 6) | BM patients only | Arm A: Afatinib | Total N in group = 30. **Total patients with Grade >3 events (only frequency >5% reported) = 10 (33%).** Rash (6), diarrhea (1), hypokalemia (1). |
|  | BM patients only | Arm B: Cisplatin/Gemcitabine | Total N in group = 18. **Total patients with Grade >3 events (only frequency >5% reported) = 11 (61%).** Vomiting (3), anemia (5), hypokalemia (3), leukopenia (4), neutropenia (7), thrombocytopenia (3), dizziness (1), decreased neutrophils (2), decreased platelets (1), WBC decreased (2). |
| Park 2016 | Whole group | Arm A: Afatinib | Total N in group = 160. **Total Grade 3+ events = 91 (57%).** Diarrhea (20), Rash (16), stomatitis (7), paronychia (3), fatigue (9), decreased appetite (1), nausea (2), nasal dryness (1), hand-foot syndrome (1), weight decreased (1), hypokalemia (3), toxic skin eruption (1), dehydration (3), pneumonia (1), confused state (1), AKI (2), skin infection (1), acidosis (1), hypercreatinaemia (1), neutropenia (1), hypoalbuminemia (1), paraneoplastic encephalomyelitis (1), flushing (1), GI pain (1), blood bicarb decreased (1), intertrigo (1), scab (1) |
|  | Whole group | Arm B: Gefitinib | Total N in group = 159. **Total Grade 3+ events = 83 (52%).** Diarrhea (2), rash (5), paronychia (1), vomiting (1), increased AST/ALT(14), conjunctivitis (1), increased aminotransferases (1), renal failure (1), interstitial lung disease (3), anal hemorrhage (1), bone marrow failure (1), hepatic failure (1), hepatitis (1) |
| Hosomi 2020 | Whole group | Arm A: Gefitinib | Total N in group = 171. **Total Grade 3+ treatment-related events = 53 (31%).** Liver dysfunction (38), leukopenia (1), neutropenia (1), anemia (4), blood bilirubin increase (1), hyponatremia (1), diarrhea (2), vomiting (1), rash (5), nail changes (2), anorexia (2) |
|  | Whole group | Arm B: Gefitinib + chemo (pemetrexed and carboplatin) | Total N in group = 170. **Total Grade 3+ treatment-related events = 111** (**65.3%).** Leukopenia (36), neutropenia (53), anemia (36), thrombocytopenia (29), liver dysfunction (21), hyponatremia (5), diarrhea (7), vomiting (4), stomatitis (1), rash (7), nail changes (5), anorexia (12), limb edema (3), fatigue (7) |
| Saito 2019 | Whole group | Arm A: Erlotinib + Bevacizumab | Total N in group = 112. **Total Grade 3 or 4 events - 98 (88%).** Rash (23), Diarrhea (6), Proteinuria (8), Non-pulmonary hemorrhage (2), hypertension (26), increased aminotransferase (9), stomatitis (1), paronychia (2), decreased appetite (2), anemia (4), gastrointestinal perforation (1), increased amylase (1), hypernatremia (1), neutropenia (1), febrile neutropenia (1), sepsis (1), strangulation ileus(1), lower limb edema (1), acute renal failure (1), skin infection (1), headache (1), thrombosis (1), decreased performance status (1), pharyngalgia (1), dacryocystitis (1) |
|  | Whole group | Arm B: Erlotinib alone | Total N in group = 114. **Total Grade 3 or 4 events- 53 (46%)**. Rash (24), Diarrhea (2), proteinuria (1), non-pulmonary hemorrhage(1), hypertension (1), increased amino transferase (6), stomatitis (1), paronychia (3), decreased appetite (1), increased amylase (1), neutropenia (1), lower limb edema (1), acute renal failure (1), skin infection (1), increased alkaline phosphatase (2), thrombosis (1), peripheral sensory neuropathy (1), hearing impaired (1), hypokalemia (1), hypophosphatemia (1), urinary retention (1) |
| Norohna 2020 | Whole group | Arm A: Gefitinib | Total N in group = 176. **Total Grade 3 or 4 events** **- 84 (49.4%).** Anemia (2), non-neutropenic infection (10), nausea/vomiting (3), fatigue (5), diarrhea (14), hypertension (40), weight loss (5), skin rash (8), dry skin (1), paronychia (1), interstitial pneumonitis (3), renal dysfunction (1), thromboembolism (2), hyponatremia (27), hypokalemia (2), hyperkalemia (1), hypomagnesemia (1), hypocalcaemia (1), elevated aminotransferase (5) |
|  | Whole group | Arm B: Gefitinib + Chemo (pemetrexed and carboplatin) | Total N iin group = 174. **Total Grade 3 or 4 events** **- 123 (75%).** Anemia (32), neutropenia (26), thrombocytopenia (8), febrile neutropenia (17), non-neutropenic infection (18), nausea/vomiting (9), anorexia (2), fatigue (8), diarrhea (22), mucositis (2), hiccups (1), hypertension (43), weight loss (6), giddiness (1), ear inflammation (1), skin rash (7), skin ulceration (1), hemoptysis (1), interstitial pneumonitis (1), renal dysfunction (10), thromboembolism (3), hyponatremia (39), hypokalemia (13), hyperkalemia (2), hypomagnesemia (1), hypocalcaemia (5), elevated aminotransferase (8) |

**Supplement C**

Several figures below contain P-scores showing ranking of treatments for each comparison. A higher P score indicates a superior treatment in the analysis (i.e. a greater relative survival benefit).


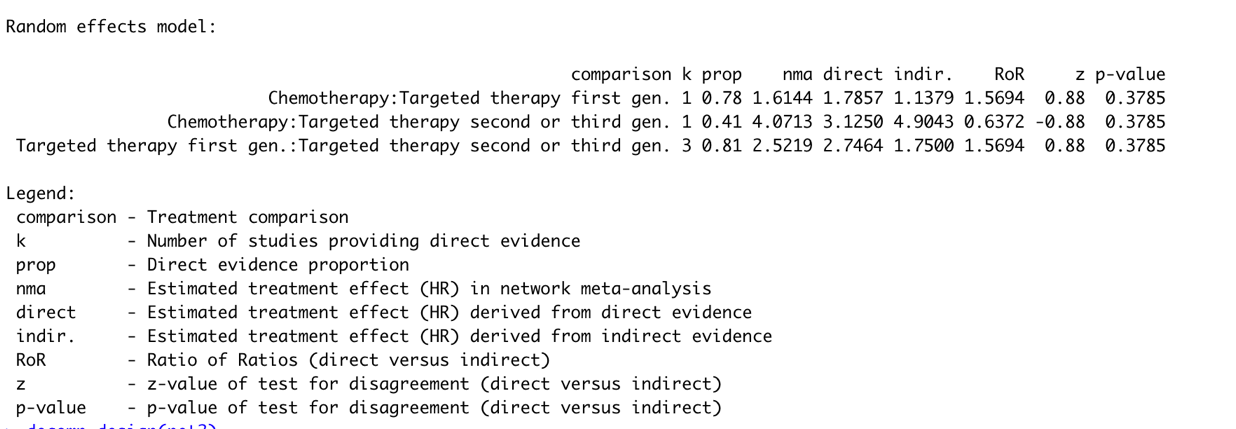


Figure S1: Targeted Therapy vs. Conventional Chemotherapy, iPFS analysis showing direct and indirect estimates

**
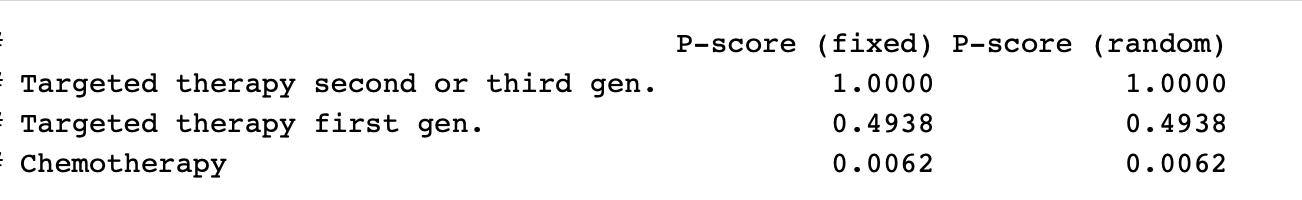
**

Figure S2: Targeted therapy vs. conventional chemotherapy in NSCLC with BMs, ranking of treatments in improving iPFS


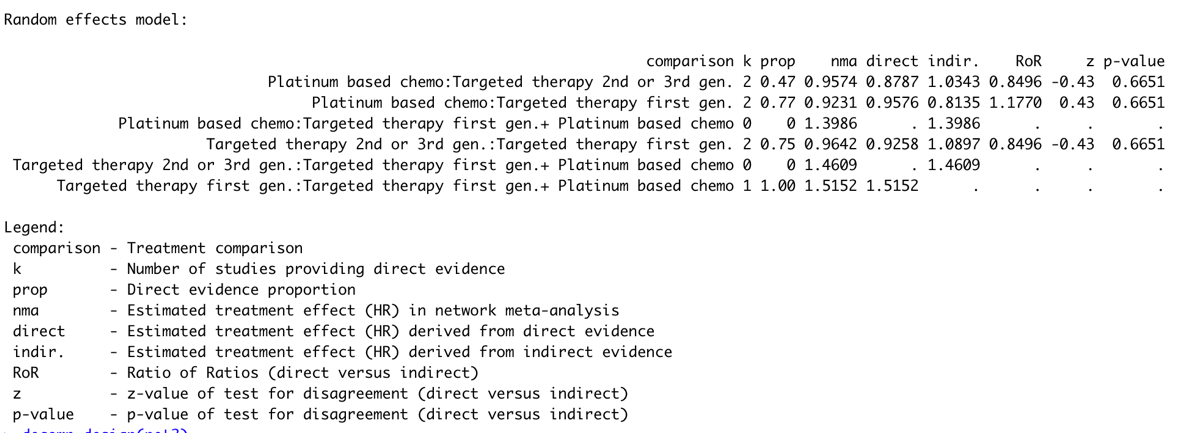


Figure S3: Targeted Therapy vs. Conventional Chemotherapy, OS analysis showing direct and indirect estimates


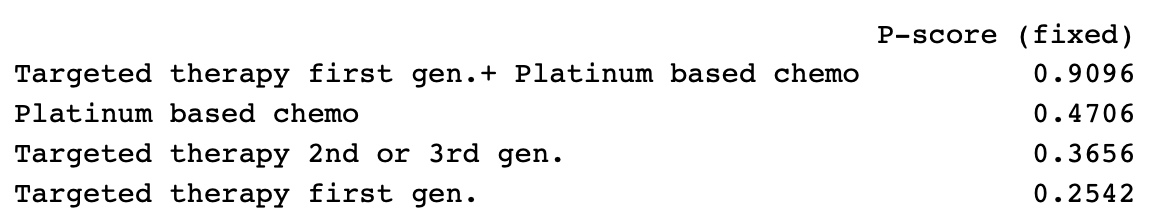


Figure S4: Targeted therapy vs. conventional chemotherapy in NSCLC with BMs, ranking of treatments in improving OS


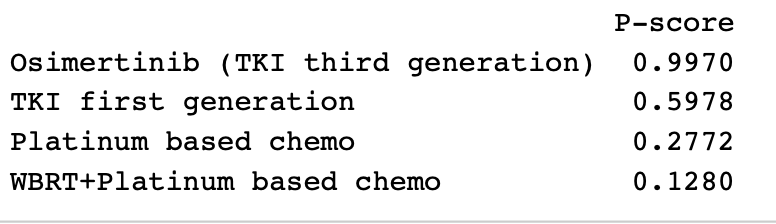


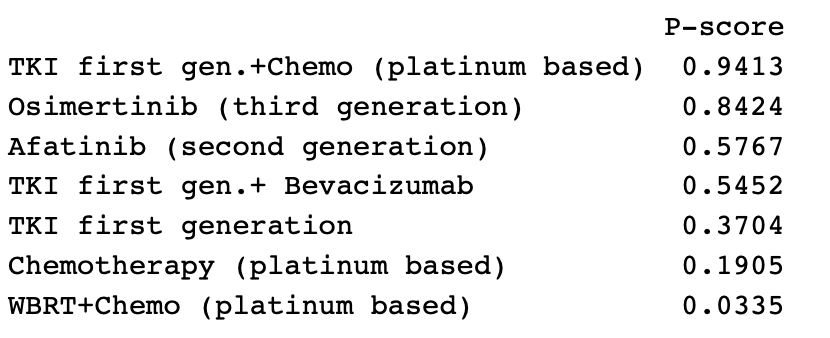
Figure S5: EGFR-mutant NSCLC with BMs, ranking of treatments in improving CNS PFS

Figure S6: EGFR-mutant NSCLC with BMs, ranking of treatments in improving Overall PFS


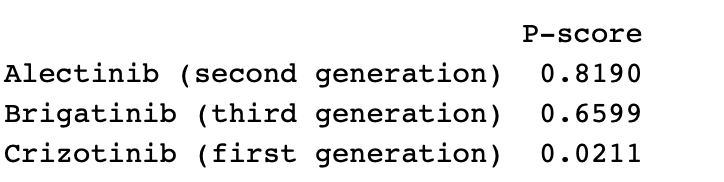


Figure S7: ALK-mutant NSCLC with BMs, ranking of treatments in improving CNS PFS


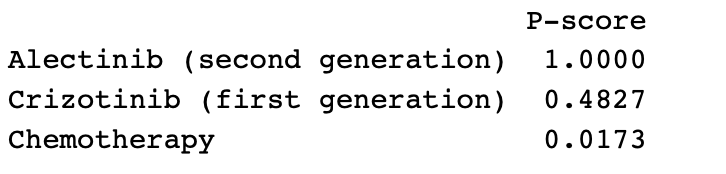


Figure S8: ALK-mutant NSCLC with BMs, ranking of treatments in improving CNS TTP


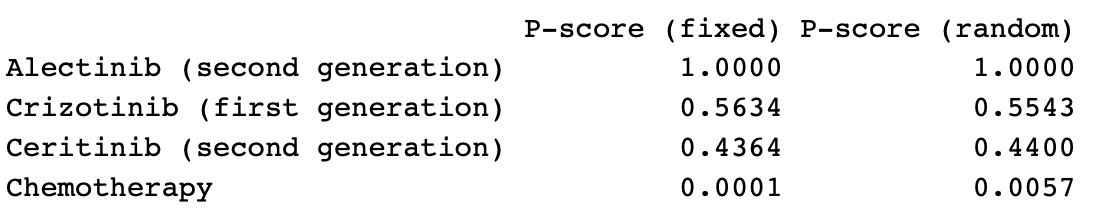


Figure S9: ALK-mutant NSCLC with BMs, ranking of treatments in improving Overall PFS

**Supplement D**

GRADE-based certainty of evidence results for each analysis, using the CINeMA framework

Table S3 – Targeted therapy vs. traditional chemotherapy for iPFS

| **Comparison** | **Number of studies** | **Within-study bias** | **Reporting bias** | **Indirectness** | **Imprecision** | **Heterogeneity** | **Incoherence** | **Confidence rating** |
| --- | --- | --- | --- | --- | --- | --- | --- | --- |
| **Mixed Evidence** |  |  |  |  |  |  |  |  |
| Chemotherapy:Targeted_therapy_first_gen | 1 | No concerns | Undetected | No concerns | No concerns | Major concerns | No concerns | Moderate |
| Chemotherapy:Targeted_therapy_second_or_third_gen | 1 | No concerns | Undetected | No concerns | No concerns | No concerns | No concerns | High |
| Targeted_therapy_first_gen:Targeted_therapy_second_or_third_gen | 3 | No concerns | Undetected | No concerns | No concerns | No concerns | No concerns | High |
| **Indirect Evidence** |  |  |  |  |  |  |  |  |

Table S4 – Targeted therapy vs. traditional chemotherapy for overall PFS (traditional meta-analysis, traditional GRADE framework used)

| **Comparison** | **Number of Studies** | **Downgrading** | | | | | **Upgrading** | | | **Confidence rating** |
| --- | --- | --- | --- | --- | --- | --- | --- | --- | --- | --- |
|  |  | Risk of Bias | Inconsistency | Indirectness | Imprecision | Publication Bias | Magnitude of Effect | Dose-Response | Potential confounding |  |
| Targeted Therapy:Chemotherapy | 9 | No concerns | No concerns | No concerns | No concerns | No concerns | N/A | N/A | N/A | High |

Table S5 - Targeted therapy vs. traditional chemotherapy for overall survival

| **Comparison** | **Number of studies** | **Within-study bias** | **Reporting bias** | **Indirectness** | **Imprecision** | **Heterogeneity** | **Incoherence** | **Confidence rating** |
| --- | --- | --- | --- | --- | --- | --- | --- | --- |
| **Mixed Evidence** |  |  |  |  |  |  |  |  |
| Platinum_chemo:Targeted_therapy_first_gen | 2 | No concerns | Undetected | No concerns | Major concerns | No concerns | No concerns | Moderate |
| Platinum_chemo:Targeted_therapy_second_or_third_gen | 2 | No concerns | Undetected | No concerns | Major concerns | No concerns | No concerns | Moderate |
| Targeted_therapy_first_gen:Targeted_therapy_first_gen+platinum_chemo | 1 | Some concerns | Undetected | No concerns | Major concerns | No concerns | No concerns | Moderate |
| Targeted_therapy_first_gen:Targeted_therapy_second_or_third_gen | 2 | No concerns | Undetected | No concerns | Major concerns | No concerns | No concerns | Moderate |
| **Indirect Evidence** |  |  |  |  |  |  |  |  |
| Platinum_chemo:Targeted_therapy_first_gen+platinum_chemo | 0 | Some concerns | Undetected | No concerns | Major concerns | No concerns | No concerns | Moderate |
| Targeted_therapy_first_gen+platinum_chemo:Targeted_therapy_second_or_third_gen | 0 | Some concerns | Undetected | No concerns | Major concerns | No concerns | No concerns | Moderate |

Table S6 –iPFS in EGFR mutated NSCLC with BMs

| **Comparison** | **Number of studies** | **Within-study bias** | **Reporting bias** | **Indirectness** | **Imprecision** | **Heterogeneity** | **Incoherence** | **Confidence rating** |
| --- | --- | --- | --- | --- | --- | --- | --- | --- |
| **Mixed Evidence** |  |  |  |  |  |  |  |  |
| Osimertinib:Platinum_Chemo | 1 | No concerns | Undetected | No concerns | No concerns | Major concerns | Major concerns | Low |
| Osimertinib:TKI_First_Gen | 1 | No concerns | Undetected | No concerns | No concerns | Major concerns | Major concerns | Low |
| TKI_First_Gen:WBRT+Platinum_Chemo | 1 | No concerns | Undetected | No concerns | No concerns | Major concerns | Major concerns | Low |
| **Indirect Evidence** |  |  |  |  |  |  |  |  |
| Osimertinib:WBRT+Platinum_Chemo | 0 | No concerns | Undetected | No concerns | No concerns | Major concerns | Major concerns | Low |
| Platinum_Chemo:TKI_First_Gen | 0 | Some concerns | Undetected | No concerns | Major concerns | No concerns | Major concerns | Low |
| Platinum_Chemo:WBRT+Platinum_Chemo | 0 | No concerns | Undetected | No concerns | Major concerns | No concerns | Major concerns | Low |

Table S7 – Overall PFS in EGFR mutated NSCLC with BMs

| **Comparison** | **Number of studies** | **Within-study bias** | **Reporting bias** | **Indirectness** | **Imprecision** | **Heterogeneity** | **Incoherence** | **Confidence rating** |
| --- | --- | --- | --- | --- | --- | --- | --- | --- |
| **Mixed Evidence** |  |  |  |  |  |  |  |  |
| Afatinib:Platinum_Chemo | 2 | Some concerns | Undetected | No concerns | No concerns | Major concerns | Major concerns | Low |
| Afatinib:TKI_First_Gen | 1 | No concerns | Undetected | No concerns | Major concerns | No concerns | Major concerns | Low |
| Osimertinib:TKI_First_Gen | 1 | No concerns | Undetected | No concerns | No concerns | Major concerns | Major concerns | Low |
| TKI_First_Gen:TKI_First_Gen+Bevacizumab | 1 | No concerns | Undetected | No concerns | Major concerns | No concerns | Major concerns | Low |
| TKI_First_Gen:TKI_First_Gen+Platinum_Chemo | 2 | Some concerns | Undetected | No concerns | No concerns | Major concerns | Major concerns | Low |
| TKI_First_Gen:WBRT+Platinum_Chemo | 1 | No concerns | Undetected | No concerns | No concerns | Major concerns | Major concerns | Low |
| **Indirect Evidence** |  |  |  |  |  |  |  |  |
| Afatinib:Osimertinib | 0 | No concerns | Undetected | No concerns | Major concerns | No concerns | Major concerns | Low |
| Afatinib:TKI_First_Gen+Bevacizumab | 0 | No concerns | Undetected | No concerns | Major concerns | No concerns | Major concerns | Low |
| Afatinib:TKI_First_Gen+Chemotherapy | 0 | No concerns | Undetected | No concerns | Major concerns | No concerns | Major concerns | Low |
| Afatinib:WBRT+Chemotherapy | 0 | No concerns | Undetected | No concerns | No concerns | Major concerns | Major concerns | Low |
| Chemotherapy:Osimertinib | 0 | No concerns | Undetected | No concerns | No concerns | Major concerns | Major concerns | Low |
| Chemotherapy:TKI_First_Gen | 0 | Some concerns | Undetected | No concerns | Major concerns | No concerns | Major concerns | Low |
| Chemotherapy:TKI_First_Gen+Bevacizumab | 0 | No concerns | Undetected | No concerns | Major concerns | No concerns | Major concerns | Low |
| Chemotherapy:TKI_First_Gen+Chemotherapy | 0 | Some concerns | Undetected | No concerns | No concerns | Major concerns | Major concerns | Low |
| Chemotherapy:WBRT+Chemotherapy | 0 | No concerns | Undetected | No concerns | Major concerns | No concerns | Major concerns | Low |
| Osimertinib:TKI_First_Gen+Bevacizumab | 0 | No concerns | Undetected | No concerns | Major concerns | No concerns | Major concerns | Low |
| Osimertinib:TKI_First_Gen+Chemotherapy | 0 | No concerns | Undetected | No concerns | Major concerns | No concerns | Major concerns | Low |
| Osimertinib:WBRT+Chemotherapy | 0 | No concerns | Undetected | No concerns | No concerns | Major concerns | Major concerns | Low |
| TKI_First_Gen+Bevacizumab:TKI_First_Gen+Chemotherapy | 0 | No concerns | Undetected | No concerns | Major concerns | No concerns | Major concerns | Low |
| TKI_First_Gen+Bevacizumab:WBRT+Chemotherapy | 0 | No concerns | Undetected | No concerns | No concerns | Major concerns | Major concerns | Low |
| TKI_First_Gen+Chemotherapy:WBRT+Chemotherapy | 0 | No concerns | Undetected | No concerns | No concerns | Major concerns | Major concerns | Low |

Table S8 – Overall survival in EGFR mutated NSCLC with BMs

| **Comparison** | **Number of studies** | **Within-study bias** | **Reporting bias** | **Indirectness** | **Imprecision** | **Heterogeneity** | **Incoherence** | **Confidence rating** |
| --- | --- | --- | --- | --- | --- | --- | --- | --- |
| **Mixed Evidence** |  |  |  |  |  |  |  |  |
| Afatinib:First_gen_TKI | 1 | No concerns | Undetected | No concerns | Major concerns | No concerns | Major concerns | Low |
| Afatinib:Platinum_chemo | 2 | Some concerns | Undetected | No concerns | Major concerns | No concerns | Major concerns | Low |
| First_gen_TKI:First_gen_TKI+Platinum_Chemo | 1 | Some concerns | Undetected | No concerns | Major concerns | No concerns | Major concerns | Low |
| First_gen_TKI:Osimertinib | 1 | No concerns | Undetected | No concerns | Major concerns | No concerns | Major concerns | Low |
| First_gen_TKI:WBRT+Platinum_chemo | 1 | No concerns | Undetected | No concerns | Major concerns | No concerns | Major concerns | Low |
| **Indirect Evidence** |  |  |  |  |  |  |  |  |
| Afatinib:First_gen_TKI+Platinum_Chemo | 0 | Some concerns | Undetected | No concerns | Major concerns | No concerns | Major concerns | Low |
| Afatinib:Osimertinib | 0 | No concerns | Undetected | No concerns | Major concerns | No concerns | Major concerns | Low |
| Afatinib:WBRT+Platinum_chemo | 0 | No concerns | Undetected | No concerns | Major concerns | No concerns | Major concerns | Low |
| First_gen_TKI:Platinum_chemo | 0 | Some concerns | Undetected | No concerns | Major concerns | No concerns | Major concerns | Low |
| First_gen_TKI+Platinum_Chemo:Osimertinib | 0 | Some concerns | Undetected | No concerns | Major concerns | No concerns | Major concerns | Low |
| First_gen_TKI+Platinum_Chemo:Platinum_chemo | 0 | Some concerns | Undetected | No concerns | Major concerns | No concerns | Major concerns | Low |
| First_gen_TKI+Platinum_Chemo:WBRT+Platinum_chemo | 0 | Some concerns | Undetected | No concerns | Major concerns | No concerns | Major concerns | Low |
| Osimertinib:Platinum_chemo | 0 | No concerns | Undetected | No concerns | Major concerns | No concerns | Major concerns | Low |
| Osimertinib:WBRT+Platinum_chemo | 0 | No concerns | Undetected | No concerns | Major concerns | No concerns | Major concerns | Low |
| Platinum_chemo:WBRT+Platinum_chemo | 0 | No concerns | Undetected | No concerns | Major concerns | No concerns | Major concerns | Low |

Table S9 – iPFS in ALK rearranged NSCLC with BMs

| **Comparison** | **Number of studies** | **Within-study bias** | **Reporting bias** | **Indirectness** | **Imprecision** | **Heterogeneity** | **Incoherence** | **Confidence rating** |
| --- | --- | --- | --- | --- | --- | --- | --- | --- |
| **Mixed Evidence** |  |  |  |  |  |  |  |  |
| Alectinib:Crizotinib | 1 | No concerns | Undetected | No concerns | Major concerns | No concerns | Major concerns | Low |
| Brigatinib:Crizotinib | 1 | Some concerns | Undetected | No concerns | No concerns | Major concerns | Major concerns | Low |
| **Indirect Evidence** |  |  |  |  |  |  |  |  |
| Alectinib:Brigatinib | 0 | Some concerns | Undetected | No concerns | Major concerns | No concerns | Major concerns | Low |

Table S10 – iTTP in ALK rearranged NSCLC with BMs

| **Comparison** | **Number of studies** | **Within-study bias** | **Reporting bias** | **Indirectness** | **Imprecision** | **Heterogeneity** | **Incoherence** | **Confidence rating** |
| --- | --- | --- | --- | --- | --- | --- | --- | --- |
| **Mixed Evidence** |  |  |  |  |  |  |  |  |
| Alectinib:Chemotherapy | 1 | No concerns | Undetected | No concerns | No concerns | No concerns | No concerns | High |
| Alectinib:Crizotinib | 2 | No concerns | Undetected | No concerns | No concerns | No concerns | No concerns | High |
| Chemotherapy:Crizotinib | 2 | No concerns | Undetected | No concerns | Major concerns | No concerns | No concerns | Moderate |
| **Indirect Evidence** |  |  |  |  |  |  |  |  |

Table S11 – Overall PFS in ALK rearranged NSCLC with BMs

| **Comparison** | **Number of studies** | **Within-study bias** | **Reporting bias** | **Indirectness** | **Imprecision** | **Heterogeneity** | **Incoherence** | **Confidence rating** |
| --- | --- | --- | --- | --- | --- | --- | --- | --- |
| **Mixed Evidence** |  |  |  |  |  |  |  |  |
| Alectinib:Chemotherapy | 1 | No concerns | Undetected | No concerns | No concerns | No concerns | No concerns | High |
| Alectinib:Crizotinib | 2 | No concerns | Undetected | No concerns | No concerns | No concerns | No concerns | High |
| Ceritinib:Chemotherapy | 2 | No concerns | Undetected | Some concerns | No concerns | Major concerns | No concerns | Moderate |
| Chemotherapy:Crizotinib | 3 | No concerns | Undetected | No concerns | No concerns | Major concerns | No concerns | Moderate |
| **Indirect Evidence** |  |  |  |  |  |  |  |  |
| Alectinib:Ceritinib | 0 | No concerns | Undetected | No concerns | No concerns | No concerns | No concerns | High |
| Ceritinib:Crizotinib | 0 | No concerns | Undetected | Some concerns | Major concerns | No concerns | No concerns | Moderate |

**Supplement E - League Tables**

The hazard ratio displayed in each cell is calculated with the treatment in the top row used as the reference. HR above 1 indicates there was a greater risk of the specific event (intracranial progression, overall progression, or survival) with the corresponding treatment listed in the first column.

**Table S12 – iPFS pooled analysis – EGFR or ALKi versus conventional chemotherapy (Random effects model)**

|  | Chemotherapy | Targeted therapy first generation | Targeted therapy second or third generation |
| --- | --- | --- | --- |
| Chemotherapy |  | 1.6144 [1.063-2.452] | 4.0713 [2.485-6.671] |
| Targeted therapy first generation | 0.6194 [0.408-0.941] |  | 2.5219 [1.703-3.735] |
| Targeted therapy second or third generation | 0.2456 [0.145-0.403] | 0.3965 [0.268-0.587] |  |

Studies included: Camidge 2018, Hida 2017, Yang 2017, Wu 2018 AURA3, Soria 2018

**Table S13 – Overall survival pooled analysis – EGFR or ALKLI versus conventional chemotherapy (Random effects model)**

|  | Platinum-based chemotherapy | Targeted therapy second or third generation | Targeted therapy first generation | Targeted therapy first generation + platinum-based chemotherapy |
| --- | --- | --- | --- | --- |
| Platinum-based chemotherapy |  | 0.957 [0.662-1.384] | 0.923 [0.678-1.256] | 1.399 [0.788-2.481] |
| Targeted therapy second or third generation | 1.045 [0.723-1.510] |  | 0.964 [0.701-1.327] | 1.461 [0.819-2.607] |
| Targeted therapy first generation | 1.083 [0.796-1.475] | 1.037 [0.754-1.427] |  | 1.515 [0.935-2.456] |
| Targeted therapy first generation + platinum-based chemotherapy | 0.715 [0.403-1.268] | 0.684 [0.384-1.221] | 0.660 [0.407-1.070] |  |

Studies included: Yang 2017, Soria 2018, Schuler 2016 LUX-Lung 3, Schuler 2016 LUX-Lung 6, Park 2016 (LUX-Lung7), Solomon 2014/2016/2018 (same trial, with updated survival results), Hosomi 2020.

**Table S14 – EGFR-mutant patients only - iPFS (fixed effects model)**

|  | Osimertinib (third generation TKI) | Platinum-based chemotherapy | First generation TKI (Erlotinib or Gefitinib) | WBRT+Platinum-based chemotherapy |
| --- | --- | --- | --- | --- |
| Osimertinib (third generation TKI) |  | 0.320 [0.148-0.690] | 0.480 [0.268-0.860] | 0.269 [0.127-0.570] |
| Platinum-based chemotherapy | 3.125 [1.449-6.738] |  | 1.500 [0.572-3.935] | 0.840 [0.287-2.461] |
| First generation TKI (Icotinib, Erlotinib or Gefitinib) | 2.083 [1.163-3.733] | 0.667 [0.254-1.749] |  | 0.560 [0.345-0.900] |
| WBRT+Platinum-based chemotherapy | 3.720 [1.754-7.890] | 1.190 [0.406-3.488] | 1.786 [1.111-2.870] |  |

Studies included: Yang 2017, Wu 2018 AURA3, Soria 2018.

**Table S15 – EGFR-mutant patients only - Overall PFS (fixed effects model)**

|  | Afatinib (second generation TKI) | Platinum-based chemotherapy | Osimertinib (third generation TKI) | First-generation TKI + Bevacizumab | First generation TKI + platinum-based chemotherapy | First-generation TKI | WBRT + platinum-based chemotherapy |
| --- | --- | --- | --- | --- | --- | --- | --- |
| Afatinib (second generation TKI) |  | 0.508 [0.271-0.952] | 1.617 [0.738-3.541] | 0.974 [0.404-2.351] | 1.938 [0.917-4.098] | 0.760 [0.401-1.440] | 0.334 [0.161-0.696] |
| Platinum-based chemotherapy | 1.969 [1.051-3.688] |  | 3.183 [1.166-8.690] | 1.918 [0.650-5.658] | 3.816 [1.437-10.138] | 1.496 [0.611-3.665] | 0.658 [0.251-1.728] |
| Osimertinib (third generation TKI) | 0.618 [0.282-1.354] | 0.314 [0.115-0.858] |  | 0.603 [0.283-1.285] | 1.199 [0.659-2.181] | 0.470 [0.299-0.740] | 0.207 [0.116-0.369] |
| First-generation TKI + Bevacizumab | 1.026 [0.425-2.476] | 0.521 [0.177-1.538] | 1.660 [0.778-3.539] |  | 1.990 [0.968-4.090] | 0.780 [0.426-1.430] | 0.343 [0.170-0.694] |
| First generation TKI + platinum-based chemotherapy | 0.516 [0.244-1.091] | 0.262 [0.099-0.696] | 0.834 [0.458-1.518] | 0.503 [0.244-1.033] |  | 0.392 [0.266-0.579] | 0.172 [0.102-0.293] |
| First-generation TKI | 1.316 [0.695-2.493] | 0.668 [0.273-1.637] | 2.128 [1.351-3.350] | 1.282 [0.699-2.350] | 2.551 [1.723-3.767] |  | 0.440 [0.307-0.630] |
| WBRT + platinum-based chemotherapy | 2.990 [1.437-6.224] | 1.519 [0.579-3.987] | 4.836 [2.711-8.625] | 2.914 [1.441-5.894] | 5.797 [3.412-9.849] | 2.273 [1.587-3.254] |  |

Studies included – Yang 2017, Soria 2018, Schuler 2016 LUX-Lung 3, Schuler 2016 LUX-Lung 6, Park 2016, Hosomi 2020, Saito 2019, Noronha 2020.

**Table S16 – EGFR-mutant patients only - Overall Survival (fixed effects model)**

|  | Afatinib (second generation TKI) | Osimertinib (third generation TKI) | Platinum-based chemotherapy | First generation TKI + platinum-based chemotherapy | First-generation TKI | WBRT + platinum-based chemotherapy |
| --- | --- | --- | --- | --- | --- | --- |
| Afatinib (second generation TKI) |  | 1.398 [0.637-3.065] | 1.138 [0.666- | 1.758 [0.785-3.933] | 1.160 [0.609-2.210] | 1.079 [0.495-2.351] |
| Osimertinib (third generation TKI) | 0.716 [0.326-1.569] |  | 0.814 [0.315- | 1.258 [0.650-2.432] | 0.830 [0.530-1.300] | 0.772 [0.413-1.444] |
| Platinum-based chemotherapy | 0.879 [0.515-1.501] | 1.228 [0.475-3.177] |  | 1.544 [0.587-4.062] | 1.019 [0.441-2.356] | 0.948 [0.368-2.439] |
| First generation TKI + platinum-based chemotherapy | 0.569 [0.254-1.273] | 0.795 [0.411-1.538] | 0.648 [0.246- |  | 0.660 [0.407-1.070] | 0.614 [0.320-1.178] |
| First-generation TKI | 0.862 [0.453-1.642] | 1.205 [0.769-1.887] | 0.981 [0.425- | 1.515 [0.935-2.456] |  | 0.930 [0.601-1.440] |
| WBRT + platinum-based chemotherapy | 0.927 [0.425-2.020] | 1.296 [0.692-2.424] | 1.055 [0.410- | 1.623 [0.849-3.126] | 1.075 [0.695-1.665] |  |

Studies included – Yang 2017, Soria 2018, Schuler 2016 LUX-Lung 3, Schuler 2016 LUX-Lung 6, Park 2016 (LUX-Lung 7), Hosomi 2020.

**Table S17 – ALK-rearranged patients only - iPFS (fixed effects model)**

|  | Alectinib (second generation ALKi) | Brigatinib (third generation ALKi) | Crizotinib (first generation ALKi) |
| --- | --- | --- | --- |
| Alectinib (second generation ALKi) |  | 0.593 [0.066-5.305] | 0.160 [0.020-1.280] |
| Brigatinib (third generation ALKi) | 1.688 [0.189-15.106] |  | 0.270 [0.135-0.540] |
| Crizotinib (first generation ALKi) | 6.250 [0.781-49.998] | 3.704 [1.852-7**.**407] |  |

Studies included: Camidge 2018, Hida 2017.

**Table S18 – ALK-rearranged patients only - iTTP (fixed effects model)**

|  | Alectinib (second generation AKLi) | Chemotherapy | Crizotinib (first generation ALKi) |
| --- | --- | --- | --- |
| Alectinib (second generation AKLi) |  | 0.113 [0.062-0.204] | 0.177 [0.111-0.282] |
| Chemotherapy | 8.890 [4.902-16.122] |  | 1.570 [0.965-2.555] |
| Crizotinib (first generation ALKi) | 5.662 [3.550-9.030] | 0.637 [0.391-1.036] |  |

Studies included: Novello 2018, Peters 2017, Solomon 2014/16/18, Wu 2018 PROFILE 1029, Zhou 2019

**Table S19 – ALK-rearranged patients only - Overall PFS (random effects model)**

|  | Alectinib (second generation ALKi) | Ceritinib (second generation ALKi) | Chemotherapy | Crizotinib (first generation ALKi) |
| --- | --- | --- | --- | --- |
| Alectinib (second generation ALKi) |  | 0.227 [0.106-0.487] | 0.133 [0.074-0.239] | 0.260 [0.155-0.436] |
| Ceritinib (second generation ALKi) | 4.411 [2.055-9.471] |  | 0.587 [0.359-0.958] | 1.147 [0.607-2.168] |
| Chemotherapy | 7.516 [4.183-13.505] | 1.704 [1.043-2.782] |  | 1.954 [1.302-2.934] |
| Crizotinib (first generation ALKi) | 3.846 [2.292-6.454] | 0.872 [0.461-1.648] | 0.512 [0.341-0.768] |  |

Studies included: Novello 2018, Peters 2017, Shaw 2013, Shaw 2017, Solomon 2014/16/18, Soria 2017, Wu 2018 PROFILE 1029
